# Sexual and reproductive health needs of refugee women on Lesbos, Greece: a participatory cross-sectional study

**DOI:** 10.1136/bmjgh-2025-019240

**Published:** 2026-06-28

**Authors:** J Sherally, A Benson, C Chen, Z Alshamari, L Bar Bari, E Beshir, Z Hosseini, H Jafari, M Jafari, K Mohamed, S Mohammadyasin, F Mutemba, S Sapounas, E Karamagioli, A Terzidis, N Yaghmaei, M L J Le Mat, M van den Muijsenbergh, T van den Akker

**Affiliations:** 1 Athena Institute, VU Amsterdam, Amsterdam, The Netherlands; 2 ISGlobal, Barcelona, Spain; 3 McMaster University Faculty of Health Sciences, Hamilton, Ontario, Canada; 4 National Hellenic Public Health Organization, Athens, Greece; 5 Global Health and Disaster Medicine, National and Kapodistrian University of Athens, School of Health Sciences, Athens, Attica, Greece; 6 KIT Royal Tropical Institute, Amsterdam, The Netherlands; 7 Department of Primary and Community Care, Radboud University Medical Center, Nijmegen, Netherlands; 8 Leiden University Medical Center, Leiden, ZH, Netherlands

**Keywords:** Gender-Based Violence, Maternal health, Community-based survey, Interdisciplinary Research, Health Services Accessibility

## Abstract

**Objective:**

To describe the prevalence of common sexual and reproductive health (SRH) indicators and healthcare access among women of reproductive age residing in Closed Controlled Access Centre (CCAC) Mavrovouni on Lesbos, Greece.

**Methods:**

A household survey comprising 119 questions across eight SRH domains was completed by 247 refugee women of reproductive age residing in CCAC Mavrovouni. Nine refugee coresearchers were engaged in a participatory action research process, contributing to question development, recruitment, data collection and analysis in June and July 2023.

**Results:**

Most women reported adequate antenatal care (25/28, 89%) and healthcare-assisted births (14/14, 100%), but postpartum care was suboptimal with 47% (7/15) not accessing any services despite 63% (10/16) reporting complications. About half (56/120, 47%) of women with children were single mothers. Two women had a child die at sea. Family planning showed considerable unmet need, with only 24% (42/178) of women using modern contraception and over 25% (17/66) desiring fertility treatment but none accessing it. Despite 84% (173/207) reporting adequate access to menstrual materials, only 14% (29/207) were able to consistently alleviate pain. Of 247 women, 151 (61%) experienced gynaecological symptoms yet 68 (45%) did not access healthcare. Low screening for sexually transmitted infections (43/240, 18%) and cervical cancer (1/246, 0.4%) was reported. Of 244 women, 74% (180/244) experienced verbal, 52% (127/244) physical and 36% (87/244) sexual abuse in their lifetime. Gender-based violence was most reported in home countries, during travel and during pushbacks. 54% (133/247) of women experienced at least one pushback and 23% (57/247) reported denial of medical care. Across all domains except breastfeeding, most women (67%–91%) had not received healthcare information.

**Conclusion:**

In refugee camp Mavrovouni, there is an urgent need for comprehensive SRH services that address diverse unmet health and information needs. Strengthening responses require cocreated, tailored interventions that are both data-driven and community-informed. Simultaneously, action must be taken to eliminate pushbacks and ensure equitable healthcare access irrespective of legal status.

**WHAT IS ALREADY KNOWN ON THIS TOPIC**

- Refugee women face poorer sexual and reproductive health (SRH) outcomes than host populations, yet their healthcare needs and access to services remain inadequately understood. They are seldom producers of knowledge about their own health.

**WHAT THIS STUDY ADDS**

- This study is unique in both its scope and methodology. It represents the first comprehensive SRH analysis in a European refugee camp, highlighting unmet needs for information, postpartum care, modern contraception, fertility treatment, cervical cancer and sexually transmitted infection screening, menstrual pain relief, gynaecological care and treatment for female genital mutilation/cutting complications.
- Through its participatory approach, this study elevates the voices of refugees as coproducers of knowledge rather than just subjects of study—thereby not only strengthening the relevance, credibility and community ownership of the findings but also challenging extractive research paradigms and illustrating how participatory methodologies can reshape evidence generation in contexts marked by structural inequality.

**HOW THIS STUDY MIGHT AFFECT RESEARCH, PRACTICE OR POLICY**

- The findings call for cocreated, tailored SRH services to address unmet needs, as well as urgent action to eliminate pushbacks and ensure equitable healthcare access for displaced populations, irrespective of legal status. This study sets a precedent for participatory approaches in humanitarian research and advocates for their broader adoption.

## Introduction

 By the end of 2022, there were an estimated 35 million refugees globally, of whom more than one-third hosted in Europe, including Turkiye.^1^ One in four refugees was a woman aged 18–59 years.^1^ Moria Reception and Identification Centre (RIC) on the island of Lesbos was Europe’s largest formal refugee camp before being destroyed by fire in September 2020. In the subsequently erected Closed Controlled Access Centre (CCAC) Mavrovouni, 23% of residents at the beginning of 2023 were women.^2^

Although sexual and reproductive health (SRH) has been recognised as a human right,^3^ significant inequities persist, with disparities disproportionately affecting marginalised groups such as refugees.^4^ In humanitarian settings, providing comprehensive SRH care poses a particular challenge.^5^ Even within high-income Europe, the SRH landscape for refugees is characterised by a fragmented system of emergency services that are insufficiently tailored to lived realities.^6^ A lack of robust evidence to inform directed action may explain why health responses are failing to meet refugees’ SRH needs.^6^

Comprehensive healthcare service planning is only possible if the target population’s needs, capacities and aspirations are understood. However, SRH interventions in humanitarian settings are traditionally driven by donor preferences^7^ and research prioritisation characterised by top-down imperatives.^8^ Despite growing calls for meaningful participation,^9^ refugees remain largely excluded and tokenised in healthcare research.^10 11^ A review of 53 WHO European Region member states found that refugees and migrants are seldom coproducers of evidence regarding their health.^12^ Yet, their participation is critical: for enhancing the credibility and relevance of research,^13^ ensuring that healthcare systems address their unique needs^14^ and promoting epistemic justice.^15^ Participatory Action Research (PAR) offers a solution. Rooted in emancipatory traditions, PAR engages those whose lives are under study throughout the research process, with the explicit aim of generating knowledge that drives positive social change.^16^ Rather than ‘research on’ refugees, PAR fosters ‘research with’ refugees,^17^ challenging the top-down approaches of traditional methodologies and dismantling harmful power dynamics.^18^

To our knowledge, no SRH situation analysis has been conducted in any European refugee camp to date. To address this gap, we conducted a participatory mixed-methods study to gain a comprehensive understanding of refugee women’s SRH status, needs and access to healthcare, alongside the availability and adequacy of SRH services in CCAC Mavrovouni on Lesbos. This paper presents a descriptive analysis of the quantitative arm of the study, examining the prevalence of key SRH indicators and the extent of healthcare access among women of reproductive age (WRA). Findings are intended to strengthen existing responses, prioritise interventions, enhance interagency collaboration, guide future research and support refugee women in reclaiming their health narratives.

## Methods

### Study design

We conducted a cross-sectional survey, which included (1) demographic and displacement characteristics such as age, sexual orientation, country of origin, education level, year of departure from country of origin, experience with pushbacks (A **pushback** refers to the forcible return of refugees or migrants from a country’s borders without allowing them to apply for asylum or undergo proper legal procedures. In the waters surrounding Lesbos, this often involves individuals being intercepted at sea and sent back to Turkish territory, frequently under dangerous and inhumane conditions. Pushbacks contravene international and regional legal frameworks, such as the principle of *non-refoulement* under the 1951 Refugee Convention, which prohibits returning individuals to places where they face persecution, torture, or inhuman treatment.), family composition and duration of stay on Lesbos; and (2) health outcomes and access to information and services across eight SRH domains, namely: maternal health, family planning, sexually transmitted infections (STIs), menstrual and gynaecological health, abortion, female genital mutilation/cutting (FGM/C) and gender-based violence (GBV, defined as child marriage, verbal abuse, physical abuse and sexual abuse). Separate informed consent was actively re-established prior to each section, and each question included the option for no response.

### Patient and public involvement

The research was preceded by over 2 years of sustained engagement and relationship-building with key stakeholders on Lesbos, including refugee community members, healthcare providers and camp management. This period of groundwork was essential for fostering trust and ownership and ensuring the study would be contextually appropriate and responsive to needs. Online supplemental file A outlines the nature and scope of coresearcher and stakeholder participation and situates this study within the broader PAR framework (JS, MLJLM and MvdM are trained PAR facilitators). Please find our author reflexivity statement in online supplemental file B.

The survey was cocreated in a participatory process involving nine coresearchers with lived experience in Moria RIC and/or Mavrovouni CCAC. Coresearchers were recruited through non-governmental organisation (NGO) and refugee networks, social media channels and word-of-mouth. Following interviews by the principal investigator (PI) JS, candidates were selected based on availability, gender, literacy, fluency in English, proficiency in their community’s native language and motivation for the role. The final team comprised one Somali, one Eritrean, one Ethiopian, one Iraqi and four Afghan women and one Congolese man. Financial compensation was collectively agreed on, with each coresearcher receiving €750 per month. The team participated in a 3-week training programme covering research methodologies, foundational knowledge of each SRH domain, ethical considerations (informed consent, confidentiality), positionality, trauma-informed and non-judgemental interviewing techniques, psychological first aid, de-escalation strategies, computer skills, triage and referral pathways.

The survey was first developed using existing toolkits and literature on SRH in conflict-affected populations.^19^,^29^ The initial 232 questions were then refined over seven drafts, resulting in a final version of 119 questions (online supplemental file C). Each draft underwent extensive group review by the coresearchers. Additionally, the first and sixth versions incorporated expert feedback from MLJLM, TvdA and MvdM (online supplemental figure 1). Coresearchers were instrumental in contextualising the survey to the specific needs and disposition of the target population (table 1). Discussions around GBV, particularly, sparked significant debate. A *deep democracy* (**Deep democracy** is both a philosophy and facilitation method that emphasises hearing *all* voices within a group (including minority and dissenting opinions) to foster inclusive and effective decision-making. This approach acknowledges and works with underlying group dynamics, emotional undercurrents, and conflicts, treating them as valuable inputs rather than obstacles. (Lewisdeepdemocracy.com)) session was held to reach consensus.

**Table 1:** Key adaptations to survey due to participatory approach

Category	Adaptation	Rationale
*Content relevance*	Added questions on pushbacks, denial of care due to legal status, death of children at sea, nutrition, child marriage and toilet hygiene.	Addressed unique circumstances and priorities identified by the refugee coresearchers.
	Localised care-seeking options by including clinic names commonly recognised by respondents.	Enhanced comprehension and relevance of response options.
*Cultural sensitivity*	Adjusted question sequence, eg, avoided immediate inquiry about LGBTQI status and moved GBV section to the end of the survey.	Minimised discomfort and encouraged openness among respondents.
	Rephrased questions on gender identity and sexual orientation to a general inquiry about whether someone felt part of the LGBTQI community.	Encouraged honest reporting and minimised stigma.
	Replaced ‘to what ethnic group do you belong’ with ‘what country are you from’.	Created a safe and inclusive environment, mitigating ethnic tensions within some countries.
	Removed questions resembling asylum interrogation processes, such as country of birth or countries travelled through.	Built trust and rapport with respondents, while providing clarity on the research aims.
	Rephrased and generalised GBV questions to avoid retraumatisation.	Reduced potential harm while collecting meaningful data.
*Ethical considerations*	Ensured consent was not only obtained at the start of the survey but also at the beginning of each section, with a brief explanation of upcoming topics.	Ensured voluntary participation and avoided pressure on sensitive topics.
	Included a ‘no response’ option for each question.	Ensured respondents retained full control over their participation and facilitated quality control.

GBV, gender-based violence; LGBTQI, lesbian, gay, bisexual, transgender, queer, intersex.

The final survey was translated into Amharic, Arabic, Farsi, French, Lingala, Somali and Tigrinya (each available on request), with back-translation to English to ensure accuracy. After uploading the survey into KoboToolbox, a cycle of pilot testing among the coresearchers led to additional adjustments, mainly refining the clarity and functionality of the questionnaire.

To address inherent power hierarchies within the research team, we implemented a range of strategies to foster meaningful participation. Communication began 3 months prior to fieldwork via a WhatsApp group to establish a collaborative tone. On arrival, the team cocreated a working agreement outlining shared values and conflict-resolution practices. A dedicated session on power dynamics, using arts-based methods such as body mapping, guided visualisation and reflexive journaling (definitions provided in online supplemental file A), supported critical reflection on hierarchies between academic researchers, coresearchers and respondents. Facilitation roles were rotated during meetings, and coresearcher input meaningfully shaped decisions regarding study design, logistics, respondent reimbursement and dissemination. Material resources such as laptops, phones and internet were shared, including communal access to the research office. Equal professional expectations were upheld across the team, with care taken to avoid stereotypes or assumptions. Time spent together beyond formal research settings further contributed to trust and cohesion. To reflect the equal value placed on diverse forms of knowledge, training was cofacilitated by external experts. For example, professionals from a mental health NGO contributed technical training on psychological first aid, while a refugee community member with dance and drama expertise led a creative workshop on rapport-building with adolescents. While we recognise that power asymmetries can never be fully eliminated, these deliberate practices supported a more participatory and reflexive research process.

### Setting

Data were collected in June and July 2023. According to occupancy lists provided by the camp director in June 2023, 2165 people resided in Mavrovouni CCAC at the time, of whom 34.5% were female.

### Participants

*Eligibility criteria:* refugee WRA (15–49 years, as defined by the WHO) registered in Mavrovouni CCAC (N=567). This age group was chosen due to pronounced needs during the reproductive period.^30^

*Sample size and strategy:* for the finite camp population, we used Yamane’s formula (n=N/1+N(e²)) to calculate a sample size of 235. This sample size allowed for the measurement of multiple SRH outcomes, assuming an average prevalence of 50%, with a 95% CI and 5% margin of error. Selection of respondents from countries of origin was done proportionally to their representation within the total population of WRA in Mavrovouni CCAC through random proportionate stratified sampling.

*Recruitment process:* we obtained an anonymised household list from camp management on 30 June 2023, allocated random identification numbers to each WRA and stratified lists by country of origin. Only the PI retained a secure, password-protected file with identifiers, accessible solely for potential medical follow-up of referred cases. Coresearchers were assigned lists based on their ability to converse in the primary language of the potential respondent rather than their country of origin. English-speaking women from Sierra Leone and Uganda were allocated to JS, AB or CC. Interviews were gender-matched, except for French/Lingala speaking women, who were interviewed by the Congolese male coresearcher. This decision was informed by advice from community representatives during coresearcher recruitment, who explained that cultural norms in their community favour discussing SRH topics with men. Following the random order, respondents were contacted by physically going to their tent or container. If a woman was unreachable, declined participation or had been relocated, the next eligible respondent from the list was approached until the target sample size for each country had been reached. Interviews were either conducted immediately or scheduled based on the woman’s availability and preference.

### Data collection

Data were collected by the interviewer using smartphones or laptops, involving sequential questioning via KoboToolbox. Interviews were conducted in private, either in the respondent’s home or a designated research space. No compensation or participatory incentives were offered. The interviewer introduced themselves and the study to each respondent, explaining its purpose and significance. They clarified the research team’s inability to provide medical care or influence asylum procedures, although referrals to healthcare services were possible when needed. Emphasis was placed on voluntary participation, with guarantees of confidentiality, secure data storage and the right to skip (‘no response’ was read out as an option for each question) or withdraw answers without consequence. Oral informed consent was obtained prior to each survey section. For adolescents aged 15–17, assent was provided by the respondent, and consent by her guardian. Feedback on the participation experience was elicited informally after each interview and documented in the interview notes.

Thorough assessment of available healthcare and referral systems was conducted prior to data collection, including mapping of healthcare facilities within and outside camp, documenting services offered, identifying referral pathways and consulting healthcare providers and camp management to understand operational constraints and access barriers. Meetings with relevant service providers ensured their awareness of the study and preparedness for a potential increase in patient referrals. Coresearchers were trained in triage and consulted the PI JS (a medical doctor with clinical and medical coordination experience on Lesbos) on any necessary referrals. Urgent cases (eg, suicidal ideation, domestic violence, unintended pregnancy) were referred immediately by phone, while less urgent cases were provided referral slips without confidential information.

To maintain data quality and ensure patient safety, each interview underwent postsession validation for consistency, accuracy and appropriate triage by AB, CC or JS. Support and accountability were fostered by daily team debriefings, group mental health support sessions (focused on self-care and management of vicarious trauma), individual wellness checks and anonymous access to a psychologist.

### Variables

Online supplemental file D provides a list defining all variables.

### Bias

*Information bias:* to mitigate interviewer bias (when the interviewer’s tone, phrasing or expectations influence responses), the team was trained on good research practices, interviewing techniques and standardised data collection procedures. Additionally, systematic validation of every survey response was conducted after each interview. Where responses were ambiguous or misinterpreted, coresearchers revisited the respondents to clarify and correct their answers. Additionally, the strong rapport established between coresearchers and their respective communities was predicted to minimise social desirability bias (the tendency of respondents to answer in a manner they believe is more acceptable or favourable).

*Selection bias:* despite employing random sampling, the potential for selection bias stemming from the political context surrounding the asylum process was recognised. For instance, during our data collection period, many new Somali arrivals were fast-tracked for transfer to mainland Greece, potentially causing an overrepresentation of respondents who had received multiple asylum rejections and had lived in camp longer. As a correction practice, camp sections primarily housing new arrivals were included in recruitment. Similarly, we hypothesised that women absent from their residences during recruitment might represent a more educated and informed demographic, possibly engaging in voluntary roles or social networks and services. To address this potential bias, we made three attempts to visit tents outside of standard office hours or utilised the coresearchers’ personal networks, before proceeding to the next individual on the household list.

### Data analysis

Data retrieval involved downloading an Excel file from the KoboToolbox web application. Data were analysed using R, employing descriptive methods. For all non-parametric data, the median and IQR (IQR, p25–p75) were calculated. Qualitative variables were summarised using frequencies and percentages. Although the study was primarily designed and powered for descriptive analysis, we conducted a limited number of exploratory Pearson χ^2^ tests to examine subgroup differences. A p value of <0.05 was considered statistically significant. Variables were chosen based on documented geographical and cultural differences and their potential to inform more tailored service provision. Specifically, we examined associations between country of origin and contraceptive use/preference and FGM/C prevalence.

Missing data were not systematically linked to any demographic or outcome and were, therefore, treated as missing at random. Where applicable, analyses were performed on available data, and no imputation was applied. Proportions of no-responses and missing data were calculated.

As part of the data analysis process, JS, AB, CC, ZA, LBB, EB, ZH, HJ, MJ, KM, SM and FM participated in a full-day sense-making session in which preliminary results were presented and systematically discussed. Coresearchers helped clarify ambiguous responses, identified possible areas of underreporting (particularly on sensitive topics) and provided context-specific explanations for patterns or inconsistencies in the data.

## Results

### Participants

While the target sample size was calculated as 235, we surveyed 252 respondents. This was because each coresearcher worked from a stratified list that included a 10% non-response margin. As recruitment was conducted independently, the overall number slightly exceeded the target before further inclusion was halted. Of the 252 respondents surveyed, five were excluded: two were over 49 years of age, two coresearchers interviewed each other, one was a coresearcher’s mother. The latter three were excluded to prevent the potential introduction of bias.

### Descriptive demographic and displacement characteristics

Sociodemographic characteristics are presented to contextualise the study population. In total, 247 WRA with a median age of 24 years (IQR: 21–29.5) were included (table 2). Respondents were primarily from Afghanistan (n=86, 34.8%), Eritrea (n=67, 27.1%) and Somalia (n=30, 12.1%) and predominantly Muslim (n=183, 74.1%) and Christian (n=61, 24.7%). Education levels were diverse with 29.6% (n=73) never having attended school, 30.8% (n=76) having attended primary school, 17.8% (n=44) having attended secondary school and 20.6% (n=51) having attended education after secondary school. Among 246 respondents, one woman self-identified as Lesbian, Gay, Bisexual, Transgender or Queer (LGBTQ+). Most respondents were single or did not cohabitate with a partner (n=157, 63.6%). The median number of children per woman was 1 (n=239, IQR: 0–3, range: 0–13). The median estimated time displaced from country of origin was 2 years (n=246, IQR: 1–5, range: 0–32) and the median length of stay at Mavrovouni CCAC was 4 months (n=247, IQR: 2–7, range: 0–58). Over half (n=133, 53.8%) had experienced at least one pushback before reaching Lesbos, with a median of two pushbacks per person (n=132, IQR: 1–4, range: 1–42). Of all respondents, 27.9% (n=69) had received a negative response to their asylum request. While most women had never been denied medical care because of their legal status (n=187, 75.7%), 23.1% (n=57) reported that they had been denied healthcare to some extent: 3.2% (n=8) always, 5.7% (n=14) often, 9.7% (n=24) sometimes and 4.5% (n=11) rarely.

**Table 2:** Demographic and displacement characteristics

Characteristics	Response, n (%)
Age in years, median (p25–p75) (n=247)	24 (21–29.5)
Age groups in years (n=247, m=0)	
15–24	125 (50.6)
25–34	90 (36.4)
35+	32 (13.0)
No response	0 (0.0)
Country of origin (n=247, m=0)	
Afghanistan	86 (34.8)
Eritrea	67 (27.1)
Somalia	30 (12.1)
Yemen	17 (6.9)
Palestine	13 (5.3)
Democratic Republic of Congo	12 (4.9)
Syria	6 (2.4)
Sierra Leone	5 (2.0)
Other (Sudan, Ethiopia, Cameroon, Iraq, Uganda, Iran, Guinea, Liberia, Tajikistan)	11 (4.5)
No response	0 (0.0)
Religion (n=247, m=0)	
Muslim	183 (74.1)
Christian	61 (24.7)
Kimbanguism	3 (1.2)
No response	0 (0.0)
Highest level of education (n=247, m=0)	
Never attended school	73 (29.6)
Primary	76 (30.8)
Secondary	44 (17.8)
Post-secondary	51 (20.6)
Other (did not finish primary school, self-taught)	3 (1.2)
No response	0 (0.0)
Identifies as LGBTQ+ (n=246, m=1)	
Yes	1 (0.4)
No	245 (99.6)
No response	0 (0.0)
Partner cohabitation (n=247, m=0)	
Yes	90 (36.4)
No	157 (63.6)
No response	0 (0.0)
Number of children, median (IQR, range) (n=239, m=1). *Note: 131 women had children. The 108 women who did not have any children were included in the ‘median number of children’ calculation.*	1 (0–3, 0–13)
No response	0 (0.0)
Number of years displaced, median (IQR, range) (n=247, m=1)	2 (1-5, 0-32)
No response	0 (0.0)
Months in camp, median (IQR, range) (n=247, m=0)	4 (2-7, 0-58)
No response	0 (0.0)
Experienced pushbacks before arriving (n=247, m=0)	
Yes	133 (53.8)
No	111 (44.9)
No response	3 (1.2)
Number pushbacks experienced, median (IQR, range) (n=133, m=0)	2 (1-4, 1-42)
No response	1 (0.8)
Received negative response to asylum request (n=247, m=0)	
Yes	69 (27.9)
No	178 (72.1)
No response	0 (0.0)
Denied medical care because of legal status (n=247, m=0)	
Always	8 (3.2)
Often	14 (5.7)
Sometimes	24 (9.7)
Rarely	11 (4.5)
Never	187 (75.7)
No response	3 (1.2)

Percentages may not total exactly 100% due to rounding.

LGBTQI, lesbian, gay, bisexual, transgender, queer, intersex.

### SRH outcomes and access to healthcare

#### Information

**Figure 1:**
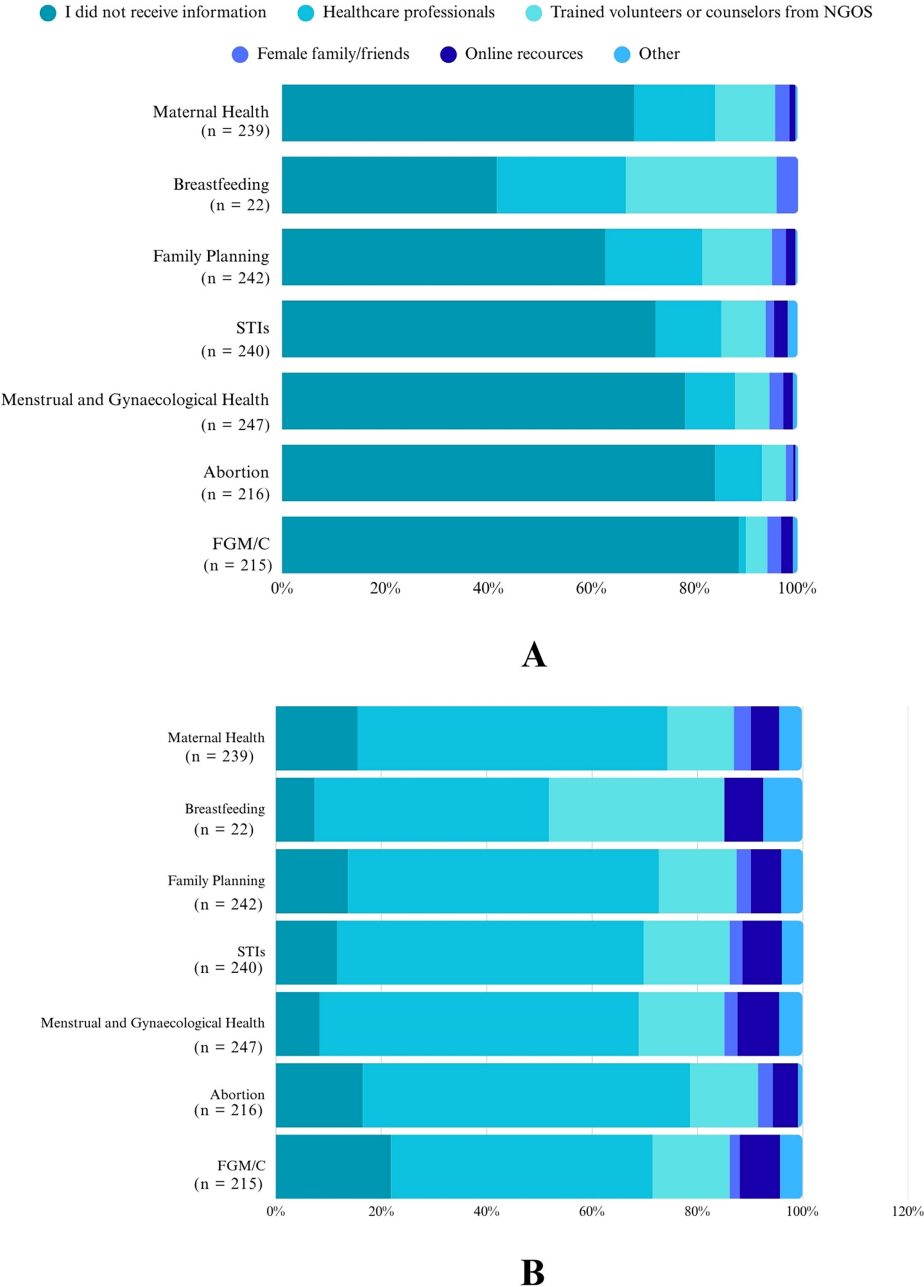
(a) Actual sources of information by SRH topic. (b) Preferred sources of information by SRH topic. Respondents could select multiple sources; for this figure, proportions of only the main reported sources are shown; ‘Other’ includes, but is not limited to: community/religious leaders, folders/pamphlets, books, school, life experience. FGM/C, female genital mutilation/cutting; NGO, non-governmental organisation; STIs, sexually transmitted infections; SRH, sexual and reproductive health.

For each SRH domain, we enquired whether women had received health information. As illustrated in figure 1a,b, for most domains, a significant majority reported not receiving information, most notably for FGM/C (n=195/215, 90.7%), abortion (n=184/216, 85.2%) and menstrual health (n=201/247, 81.4%). Breastfeeding was an exception, with a lower percentage of 45.5% (n=10/22) stating they had not received information. Healthcare professionals constituted the primary source of information across all domains except for breastfeeding and FGM/C, where trained volunteers or NGO counsellors were more commonly cited.

Women expressed a clear desire for information. The highest discrepancy between desired and received information was observed for menstrual health: while 81.4% (n=201) had not received information, only 10.5% (n=26) expressed that they did not require information. Healthcare professionals were the most commonly preferred source across all domains, with 54.5% (n=12) to 77.3% (n=191) of respondents selecting them for breastfeeding and menstrual health, respectively. Trained volunteers and NGO counsellors were the second most commonly preferred, with preferences varying between domains (eg, 40.9% (n=9) for breastfeeding and 14.8% (n=32) for abortion).

#### Maternal health

Of respondents, 97.2% (n=240) consented to answering questions on maternal health. Almost half of women with children (n=56, 46.7%) were identified as single mothers. Sixteen women (12.9%) had given birth while residing in camp and 12 (9.7%) were breastfeeding at the time of the survey. Eighteen women (7.5%, of whom four were single) were pregnant, with a mean gestational age of 4.24 months (n=18, IQR: 2–6).

Regarding adverse outcomes for in-camp pregnancies, two women reported a miscarriage before 6 months’ gestation. No stillbirths or neonatal deaths after 6 months’ gestational age, within 1 hour, or within 1 month after birth were reported. Two respondents had a child under 18 die during their (sea) journey to Lesbos.

##### Antenatal care

Among respondents who had given birth in camp or who were pregnant at the time of survey, 89.3% (n=25) had accessed antenatal care (ANC) for the first time at a median of 4.0 months into pregnancy (n=25, IQR: 1.8–7.0). The median number of ANC visits was 7 (n=27, IQR: 2–10, range: 0–28). Regarding food intake during pregnancy, 12 women (41.4%) reported not receiving food different from their usual diet, and 19 (65.5%) had not received increased food quantities.

The most accessed services were ultrasound screenings (n=26, 89.7%), (supplements like) folic acid and multivitamins (n=21, 72.4%), and blood pressure monitoring (n=21, 72.4%) (online supplemental figure 2). The least accessed services were family planning counselling (n=3, 10.3%) and mental health support (n=4, 13.8%). Most ANC was provided at health clinics located in camp (n=23, 79.3%).

Regarding complications, 20.7% (n=6) reported none, while severe morning sickness (n=14, 48.3%), anaemia (n=11, 37.9%), and urinary tract infections (n=9, 31.0%) were commonly described (online supplemental figure 3). One woman reported a uterine rupture during birth. Of those with complications, the majority had accessed care at in-camp clinics (n=18, 78.3%); one woman had not sought any care.

##### Birth

All births occurred at a hospital outside camp (n=14), with nine women giving birth vaginally and five by caesarean section. None had requested a caesarean section themselves. Among the two unplanned caesarean sections, one woman did not know why she underwent the procedure. Eight women went into labour spontaneously, while others had their labour induced due to post-term pregnancy (n=1), foetal distress (n=1), pregnancy complications (n=2), or maternal fatigue (n=1). One respondent was unsure of the reason for induction.

##### Postpartum care and breastfeeding

The median number of post-partum visits was 0.5 (n=14, IQR: 0.0–4.3, range: 0–10); seven women (46.7%) did not access postpartum services at all. Postpartum complications (online supplemental figure 4) were reported by 10 (62.5%), the most common being painful urination (n=7, 43.8%), anxiety (n=6, 37.5%), and hot, swollen, painful breasts (n=5, 31.3%). Only one woman with complications did not seek care, and most (n=7, 63.6%) sought treatment at a health clinic within camp.

Among those who completed breastfeeding, the median duration was 3 months (n=10, IQR: 1–6). Mothers who had finished breastfeeding or were still breastfeeding at the time of the survey introduced liquids other than breastmilk at a mean age of 4 months (n=14, IQR: 1–6). The median age for introducing solid food was 5 months (n=10, IQR: 2–6).

### Family planning and abortion

In total, 242 out of 247 respondents (98.0%) consented to questions regarding family planning. A significant majority - 72.7% (n=176) - expressed a desire to prevent pregnancy. Among the 66 women who did not wish to prevent pregnancy, none were undergoing fertility treatment, although 25.8% (n=17) indicated an interest in receiving such treatment. Of the 178 women who said they were using contraception, the majority relied on abstinence (n=91, 51.1%), male condoms (n=22, 12.4%) or withdrawal (n=18, 10.1%), limiting their engagement with family planning services. Among those who accessed services, most (n=25, 19.2%) obtained contraceptives from a health clinic outside camp. Twelve women (5.0%) reported using the emergency contraception pill, with repeated use (n=12, mean: 5.17, range: 1–24) among those who had accessed it.

**Figure 2:**
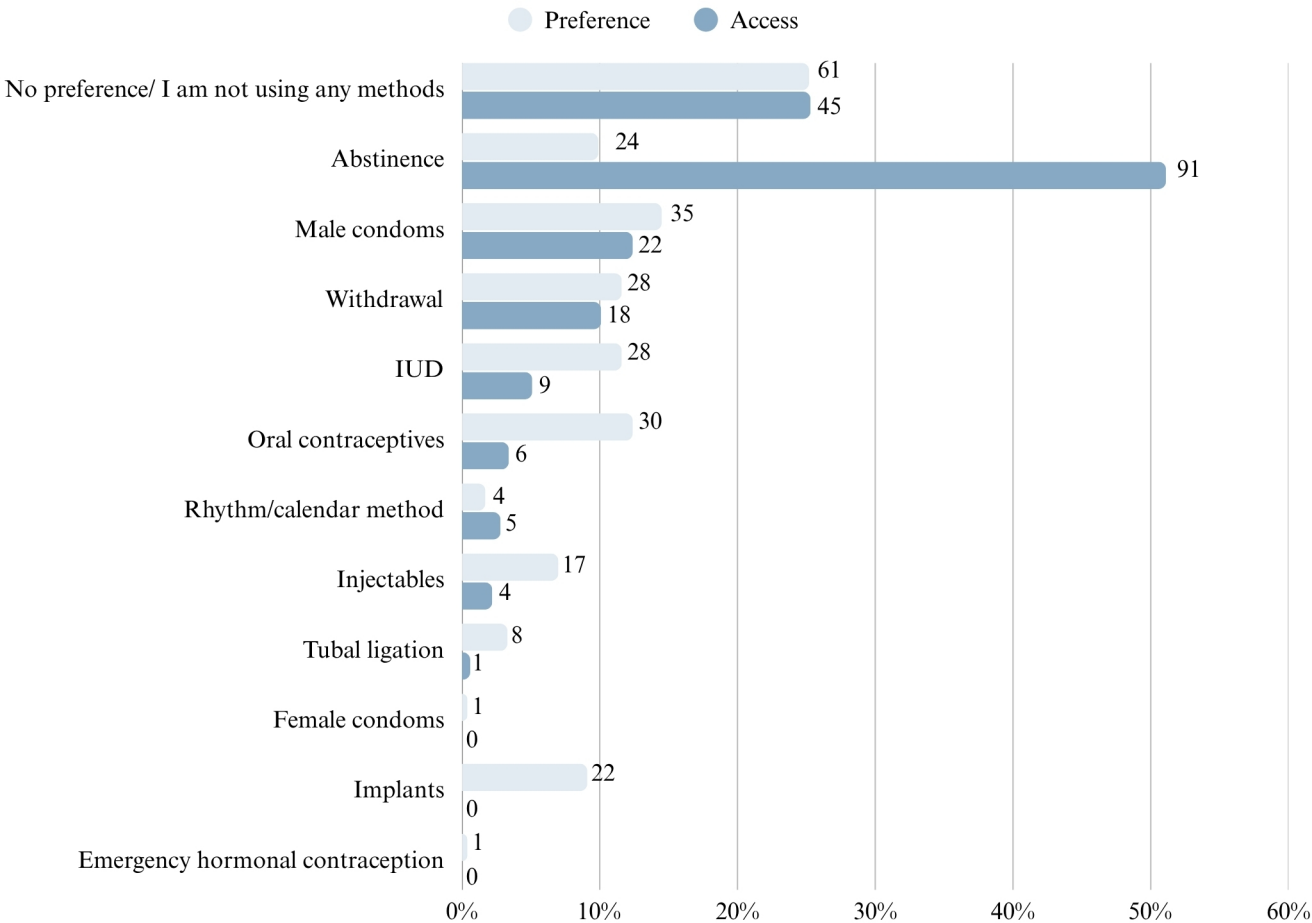
Preference for (n=242) and use of (n=178) various methods of contraception. IUD, intra-uterine device.

We observed a notable discrepancy between actual and preferred contraception method (figure 2). Modern contraceptive methods accounted for 58.7% of reported preferences (n=142), but only 23.6% of reported use (n=42). A substantial portion (n=61, 25.2%) of women did not have a preference. Bivariate analysis (online supplemental file E) comparing women who preferred modern contraceptives to those who did not, did not reveal significant differences between countries of origin (χ² = 10.19, df=5, p 0.070). However, country of origin was significantly associated to whether respondents accessed/used modern contraceptives (χ² = 48.92, df=5, p<0.001).

Among 217 respondents, two women reported having an abortion since arriving in camp, while another two reported complications from prior abortions, and three declined to respond to the question. Sensitivity surrounding the topic was evident, with 30 of 247 (12.1%) respondents declining consent to questions regarding pregnancy termination. Of the two abortions reported in camp, one was performed at 6 weeks gestation using medication from a healthcare professional, while the other, at 24 weeks, involved a surgical procedure.

### Menstrual health

Among the 247 respondents, 83.8% (n=207) reported having had a menstrual period since arriving in camp. The majority (n=173, 83.6%) had sufficient menstrual materials, with single-use menstrual pads being the most used (n=191, 92.3%) and preferred (n=213, 86.6%) material. Women primarily changed their menstrual materials in camp toilets, the conditions of which are detailed in online supplemental file C). Only 29 women (14.0%) reported being able to consistently alleviate menstrual pain, with the predominant pain relief method being home remedies or self-care techniques (n=94, 61.0%).

### Gynaecological health and STIs

All 247 respondents consented to gynaecological health questions, and 240 (97.2%) to STI-related questions. To prevent redundancy, we grouped data together. Of 246 women, only one had undergone cervical cancer screening, conducted at an in-camp health clinic. Over half of respondents (n=151, 61.1%) had experienced gynaecological symptoms since arrival, the most prevalent being itchiness (n=85, 34.4%), unusual discharge (n=77, 31.2%), and painful urination (n=70, 28.3%) (online supplemental figure 5). One in five (n=52) experienced irregular periods and 15.0% (n=37) painful sexual intercourse. Among the 151 women who experienced symptoms, care-seeking was most commonly reported at in-camp health clinics (n=66, 43.7%), followed by clinics outside camp (n=54, 35.8%), while many (n=68, 45.0%) did not seek any healthcare.

Among 240 respondents, 82.1% (n=197) had not been tested for an STI/HIV since arriving in camp. Most of the 43 tests (n=32, 74.4%) were conducted at health clinics outside camp.

### Female genital mutilation/cutting

Of the 215 women (87.0%) who consented, 76 (35.3%) reported having been impacted by FGM/C at a median age of 6.5 years (n=72, range: 0–25). The prevalence of FGM/C varied significantly by country of origin (χ² = 106.64, df=5, p<0.001) with Somalia (n=28, 96.6%) and Yemen (n=13, 76.5%) exhibiting the highest rates. No respondents from Afghanistan or Palestine reported FGM/C (online supplemental file E). Notably, 89.5% (n=68) of respondents did not seek treatment for FGM/C-related complications (e.g.: pain, psychological distress, reduced libido) while in camp.

### Gender-based violence

Among the 244 respondents (98.8%) who consented to this section, 27.6% (n=67) reported marriage before the age of 18. Additionally, 73.8% (n=180) experienced verbal abuse, 52.0% (n=127) physical abuse, and 35.7% (n=87) sexual abuse in their lifetime. All forms of GBV were most reported in respondents’ home countries, with 48.8% (n=119) of women experiencing verbal abuse, 36.1% (n=88) physical abuse, and 22.5% (n=55) sexual abuse. The other most frequent locations for GBV were during travel to Lesbos (20.1% verbal, 8.6% physical, 7.0% sexual) and pushbacks (18.4% verbal, 9.8% physical, 3.7% sexual). The most common perpetrators of verbal abuse were police or prison guards (n=88, 48.9%), and of physical abuse were family members (n=42, 33.1%) and partners (n=40, 31.5%). In contrast, sexual abuse was mainly perpetrated by neighbours or community members (n=27, 31.0%) and partners (n=26, 29.9%) (online supplemental figure 6). Notably, 80.2% (n=101) of those who reported physical abuse did not access care for their injuries, and 63.2% (n=55) of those who experienced sexual abuse did not seek care.

## Discussion

This study provides valuable insights into SRH outcomes and healthcare access of refugee WRA residing in CCAC Mavrovouni on Lesbos. We found substantial gaps in access to healthcare information, post-partum care, modern contraception, fertility treatment, cervical cancer and STI/HIV screening, menstrual pain relief, gynaecological care, and treatment for FGM/C complications. Many women reported having been denied medical care. Substantial levels of GBV occurred during travel to Lesbos and in the context of pushbacks. Notably, half of respondents had experienced at least one pushback. Finally, the high proportion of single mothers in CCAC Mavrovouni warrants further research to understand its impact on SRH outcomes and access.

These findings underscore a critical gap in ensuring SRH rights. Access to information is not only a fundamental right mandated in Sustainable Development Goal (SDG) 3.7, but also a prerequisite for informed decision-making and equitable access to healthcare services.^30^ Language barriers in particular—as observed in other European refugee camps^31^,^38^—often prevent refugees from communicating their wishes or providing informed consent,^31^,^38^ and may be further compounded by service providers’ assumptions of refugee women’s passive reproductive agency.^39^ Verbal communication has proven more effective than written materials in populations with high illiteracy rates.^32 40^ Information needs, however, should not be generalised. Instead, SRH education should be tailored to align with the culture, education, and social dynamics of each population. For example: whereas healthcare providers were the preferred information source for WRA in CCAC Mavrovouni, adolescents in refugee camps in Uganda,^41^ Ethiopia,^42^ and Thailand^42^ preferred parents or guardians.

Antenatal care (ANC) in CCAC Mavrovouni exceeded levels reported among refugees in non-camp settings in Türkiye^43^,^45^ and Lebanon and Jordan,^46^ although comparisons should consider the differing contexts and our small sample size (n=16) for ANC-related questions. Our findings, comparable to camps on the Thai-Myanmar border^47^ and in Uganda,^48^ add to the body of evidence suggesting that camp residence may positively influence ANC access. Post-partum care, however, remains a challenge across the globe,^45 49^ and in our population, the discrepancy between reported access and complications is concerning. Mental health merits particular attention, with 6 of the 16 women who had given birth in camp describing anxiety or depression after childbirth, yet mental health counselling being the least accessed service during ANC.

The modern contraceptive prevalence rate of 23.6% found in our study is far short of the SDG target of meeting 75% of family planning needs with modern methods. Notably, Greece reports one of the lowest coverage rates in Europe, at just 38%.^50^ Studies in Türkiye,^44 51 52^ the Levant,^4653^,^55^ and Uganda^56 57^ similarly report high unmet needs for modern contraception among refugees. Low coverage may result from multiple factors.^58^ For our particular population, the substantial discrepancy between preferred and actual contraceptive method was striking, with modern options favoured but traditional methods more commonly used, highlighting a gap between reproductive intentions and access. While preference for modern contraceptives did not significantly differ by country of origin, actual use did. This disparity may reflect nationality-specific barriers, but further research is needed to explore the multifactorial drivers of contraceptive use in CCAC Mavrovouni. In the qualitative arm of our study, we explore this further. Finally, our data suggest under-reporting of abortions; as also highlighted during sense-making sessions with the coresearchers and likely resulting from stigma.^59^

The high unmet need for fertility treatment in CCAC Mavrovouni is not surprising. Despite its inclusion in the 1994 ICPD definition of reproductive health, infertility remains largely neglected in global health,^30^ with large disparities between high and low-income countries.^60^ Beyond the rights-based rationale, addressing infertility is a public health imperative, as it has been associated with psychological distress,^61^ intimate partner violence,^62^ risky sexual behaviour^63^ and social repercussions such as stigma,^64^ marital instability^65^ and economic hardship^66^—all of which disproportionately affect women.^67^ While assisted reproductive technologies may be costly, women in CCAC Mavrovouni lacked access to even basic fertility services, such as initial assessments, psychological counselling and education. This gap is particularly concerning given that over 80% of women in our population were untested for STIs.

Our findings on gynaecological and menstrual health align with studies documenting high rates of gynaecological morbidity among refugee women^68^,^70^ and underscore the need for targeted interventions. While toilets in CCAC Mavrovouni were generally considered private and safe, concerns about cleanliness and a lack of soap were prevalent. Involving refugee women in WASH infrastructure planning may help address these gaps and improve menstrual health, as demonstrated in camp settings in Bangladesh^71^ and Kenya.^72^

To our knowledge, this is the first study to quantify the prevalence of pushbacks. While grey literature has qualitatively described pushbacks,^73^,^76^ and organisations such as the Turkish Coast Guard^77^ and Aegean Boat Report (reporting a total of 354 pushbacks from Lesbos in 2023)^78^ have documented incidences, no prevalence estimates exist. Over half of our respondents had experienced at least one pushback, with some reporting as many as 42, highlighting the scale of the violation. Insights from the sense-making sessions with coresearchers suggest this figure may not fully capture the true extent. These findings are particularly concerning given the risks of GBV associated with pushbacks. Our findings on lifetime prevalences of GBV exceed global estimates^79^ and align with the existing literature documenting increased physical and sexual abuse among refugees across the migration trajectory, including in countries of origin, during transit and in the host settings.^5880 80 81 81^,^86^ That almost two-thirds of women who had experienced sexual violence did not seek care points to critical gaps in healthcare access and warrants further research.

### Strengths and limitations

This study is the first comprehensive SRH analysis in a European refugee camp, providing novel insights into SHR outcomes, healthcare—including information—access, and the prevalence of pushbacks. Strengths include the cocreated, contextualised questionnaire and the stratified random sampling approach, increasing relevance and generalisability of our findings. Refugee coresearchers enabled nuanced data collection on sensitive topics, while the sense-making process enhanced the reliability of findings. Debriefing sessions and analysis of the postinterview notes revealed a highly positive participation experience and provided valuable leads for further qualitative exploration. Some respondents shared experiences they had never disclosed, even to psychologists, warranting further research into the barriers refugees face in conventional therapeutic settings. Community buy-in was strong, with camp residents eager to engage, medical actors pleased to collaborate, and camp management supportive, underscoring the project’s integrative and inclusive ethos.

Several factors contributed to the accuracy of our findings. The participatory approach fostered rapport, as coresearchers were trusted members of the community, encouraging honest responses. Comprehensive training in research ethics, survey administration and interview techniques, combined with daily debriefings and systematic validation of survey entries, supported consistent and rigorous data collection while addressing concerns around confidentiality and power dynamics. Interviews were back-translated and conducted in respondents’ preferred languages by culturally and linguistically aligned coresearchers, reducing misinterpretation. Nonetheless, we acknowledge that certain topics (such as pushbacks, abortion, sexual orientation and education status) remained sensitive and may have been under-reported despite these efforts.

Limitations were related to the fact that refugees are a highly dynamic and heterogeneous group, and the fluctuating population in CCAC Mavrovouni posed challenges for random sampling and respondent recruitment. Transfers, extreme weather and residents leaving camp necessitated logistic adjustments, such as conducting interviews later in the day and providing taxi stipends for coresearchers to travel off-site. We did not systematically record the number of women approached who declined or were unavailable to participate. As a result, we were unable to calculate a response rate, limiting our ability to assess sample representativeness. Despite application of skip logic (particularly relevant for longer modules such as maternal health), its overall length may have contributed to responder fatigue. To mitigate, we conducted interviews at the respondent’s preferred time, location and pace and observed enthusiastic participation.

The sequential design of the questionnaire limited our analysis as access to care was only assessed for respondents reporting specific healthcare needs. Moreover, we did not collect information regarding the frequency of symptoms or access. Similarly, questions about sexual activity were omitted, as these were deemed culturally inappropriate by the coresearchers, complicating the analysis of unmet contraceptive needs. We also acknowledge that reported symptoms do not equate formal medical diagnoses, highlighting the need for triangulation with other sources of data. Designed as a descriptive, participatory baseline study in a setting where SRH data and meaningful PAR are lacking, this study offers broad, detailed and community-led insights. While these foundational findings are valuable, future research using multivariable statistical methods could help identify key predictors and guide more targeted interventions.

## Conclusion

In CCAC Mavrovouni, refugee women face substantial unmet SRH needs, including access to information, postpartum care, modern contraception, fertility services, cervical cancer and STI screening, menstrual pain management, gynaecological care and treatment for complications related to FGM/C. Addressing these gaps requires cocreated, tailored SRH responses that are both data-driven and community-owned. Additionally, urgent action is needed to end pushbacks and guarantee equitable access to care irrespective of legal status. By centring refugee voices throughout the research process, this study sets a precedent for participatory approaches in humanitarian research and supports their broader integration into policy and practice.

## Supplementary material

10.1136/bmjgh-2025-019240 online supplemental figure 1

10.1136/bmjgh-2025-019240 online supplemental figure 2

10.1136/bmjgh-2025-019240 online supplemental figure 3

10.1136/bmjgh-2025-019240 online supplemental figure 4

10.1136/bmjgh-2025-019240 online supplemental figure 5

10.1136/bmjgh-2025-019240 online supplemental figure 6

10.1136/bmjgh-2025-019240 online supplemental file 1

10.1136/bmjgh-2025-019240 online supplemental file 2

10.1136/bmjgh-2025-019240 online supplemental file 3

10.1136/bmjgh-2025-019240 online supplemental file 4

10.1136/bmjgh-2025-019240 online supplemental file 5

## Data Availability

Data are available upon reasonable request.

## References

[1] United Nations High Commissioner for Refugees (2023). Global trends: forced migration in 2022.

[2] United Nations High Commissioner for Refugees (2022). Lesvos Island weekly snapshot: 31 Jan - 6 feb 2022.

[3] United Nations (1995). Report of the international conference on population and development.

[4] Casey SE (2015). Evaluations of reproductive health programs in humanitarian settings: a systematic review. Confl Health.

[5] Onyango MA, Hixson BL, McNally S (2013). Minimum Initial Service Package (MISP) for reproductive health during emergencies: Time for a new paradigm?. Glob Public Health.

[6] Sherally J, Mat ML, Mvd M (2025). Access to sexual and reproductive healthcare for refugees transiting through Europe: a scoping review.

[7] Singh NS, Ataullahjan A, Ndiaye K (2021). Delivering health interventions to women, children, and adolescents in conflict settings: what have we learned from ten country case studies?. The Lancet.

[8] MacFarlane A, Puthoopparambil SJ, Waagensen E (2023). Framework for refugee and migrant health research in the WHO European Region. Tropical Med Int Health.

[9] MacFarlane A, Huschke S, Marques MJ (2024). Normalising participatory health research approaches in the WHO European region for refugee and migrant health: a paradigm shift. *The Lancet Regional Health - Europe*.

[10] van den Muijsenbergh M, Teunissen E, van Weel-Baumgarten E (2016). Giving voice to the voiceless: how to involve vulnerable migrants in healthcare research. Br J Gen Pract.

[11] Cook T, Dias S, Madsen W (2019). Participatory Research for Health and Social Wellbeing.

[12] MacFarlane A, Ogoro M, de Freitas C (2021). Migrants’ involvement in health policy, service development and research in the WHO European Region: A narrative review of policy and practice. *Tropical Med Int Health*.

[13] Brún T, Okonkwo E, Bonsenge-Bokanga J-S (2016). Using Participatory Learning & Action research to access and engage with ‘hard to reach’ migrants in primary healthcare research. BMC Health Serv Res.

[14] WHO Regional Office for Europe (2022). Participatory Health Research with Migrants: A Country Implementation Guide.

[15] Bhakuni H, Abimbola S (2021). Epistemic injustice in academic global health. Lancet Glob Health.

[16] (ICPHR) ICfPHR (2013). Position Paper 1: What Is Participatory Health Research.

[17] World Health Organisation (2022). Participatory health research with migrants: a country implementation guide.

[18] Omodan BI, Dastile NP (2023). Analysis of Participatory Action Research as a Decolonial Research Methodology. Soc Sci.

[19] Gipson JD, Bornstein MJ, Hindin MJ (2020). Infertility: a continually neglected component of sexual and reproductive health and rights. Bull World Health Organ.

[20] Sommer M, Schmitt M, Clatworthy D (2017). A Toolkit for Integrating Menstrual Hygiene Management (MHM) into Humanitarian Response.

[21] Bell SO, OlaOlorun F, Shankar M Measurement of abortion safety using community-based surveys: findings from three countries. (1932-6203 (electronic)).

[22] Centers for Disease Control and Prevention (2022). PRAMS phase 8 topic reference document.

[23] WHO/UNICEF Joint Monitoring Programme for Water Supply SaHJ (2022). Proposed questions on menstrual health for inclusion in household survey questionnaires for individual women. https://washdata.org/sites/default/files/2023-02/jmp-2022-updated-questions-menstrual-health-hh-surveys-zero-draft.pdf.

[24] Hynes MT, Marianne E (2007). Reproductive health assessment toolkit for conflict-affected women.

[25] National Center for Chronic Disease Prevention and Health Promotion (2011). A process evaluation of the reproductive health assessment (RHA) toolkit for conflict-affected women: a report of findings, recommendations, and next steps. Health DoR.

[26] World Health Organisation (2016). WHO recommendations on antenatal care for a positive pregnancy experience.

[27] World Health Organisation (2018). Guidance on ethical considerations in planning and reviewing research studies on sexual and reproductive health in adolescents.

[28] Sikweyiya Y (2012). Ethical and Safety Recommendations for Research on Perpetration of Sexual Violence.

[29] Peterman A, Devries K, Guedes A (2023). Ethical reporting of research on violence against women and children: a review of current practice and recommendations for future guidelines. BMJ Glob Health.

[30] Starrs AM, Ezeh AC, Barker G (2018). Accelerate progress—sexual and reproductive health and rights for all: report of the Guttmacher– Lancet Commission. The Lancet.

[31] Scott HM, Wallis N (2021). Maternity care for refugees living in Greek refugee camps: What are the challenges to provision?. Birth.

[32] Malakasis CH (2016). Migrant maternity care in athens, greece, 2016-2017: a policy report.

[33] Borsari L, Stancanelli G, Guarenti L (2018). An Innovative Mobile Health System to Improve and Standardize Antenatal Care Among Underserved Communities: A Feasibility Study in an Italian Hosting Center for Asylum Seekers. J Immigrant Minority Health.

[34] Puthoopparambil SJ (2013). Assessment report malta: the health situation at eu southern borders - migrant health, occupational health, and public health.

[35] Wolfensohn G (2016). Gender assessment of the refugee and migration crisis in serbia and fyr macedonia.

[36] Women’s Refugee Commission (2016). EU-turkey agreement failing refugee women and girls.

[37] Ayhan Baser D, Mıhcı Ö, Direk MT (2021). Views and experiences of family physicians about Syrian refugee patients in Turkey: a qualitative research. *Prim Health Care Res Dev*.

[38] Žagar M, Rotar Pavlič D, Švab I (2019). Through health workers’ eyes: a qualitative study of health service provision for migrants at Schengen border. Int J Equity Health.

[39] Grotti V, Malakasis C, Quagliariello C (2018). Shifting vulnerabilities: gender and reproductive care on the migrant trail to Europe. CMS.

[40] Döner P, Şahin K (2021). “This is not my decision; I have no alternative”. Perceptions and experiences of marriage age and family planning among Syrian women and men: a primary care study. Prim Health Care Res Dev.

[41] Ivanova O, Rai M, Mlahagwa W (2019). A cross-sectional mixed-methods study of sexual and reproductive health knowledge, experiences and access to services among refugee adolescent girls in the Nakivale refugee settlement, Uganda. *Reprod Health*.

[42] Kågesten AE, Zimmerman L, Robinson C Transitions into puberty and access to sexual and reproductive health information in two humanitarian settings: a cross-sectional survey of very young adolescents from somalia and myanmar.

[43] Torun P, Mücaz Karaaslan M, Sandıklı B (2018). Health and health care access for Syrian refugees living in İstanbul. Int J Public Health.

[44] Şimşek Z, Yentur Doni N, Gül Hilali N (2018). A community-based survey on Syrian refugee women’s health and its predictors in Şanliurfa, Turkey. *Women & Health*.

[45] WHO Regional Office for Europe (2019). Survey on the health status, services utilization and determinants of health syrian refugee population in Turkey.

[46] DeJong J, Ghattas H, Bashour H (2017). Reproductive, maternal, neonatal and child health in conflict: a case study on Syria using Countdown indicators. BMJ Glob Health.

[47] Benner MT, Mohr O, Kaloy W (2024). Mother, child and adolescent health outcomes in two long-term refugee camp settings at the Thai-Myanmar border 2000–2018: a retrospective analysis. *Prim Health Care Res Dev*.

[48] Rustad SA, Binningsbø HM, Gjerløw H Maternal health care among refugees and host communities in Northern Uganda: access, quality, and discrimination.

[49] Victora CG, Requejo JH, Barros AJD (2016). Countdown to 2015: a decade of tracking progress for maternal, newborn, and child survival. *The Lancet*.

[50] (2024). European parliamentary forum for sexual and reproductive rights. https://www.epfweb.org/node/730.

[51] Coşkun AM, Özerdoğan N, Karakaya E (2020). Fertility characteristics and related factors impacting on Syrian refugee women living in Istanbul. Afr Health Sci.

[52] Özşahin A, Emre N, Edirne T (2021). Contraceptive use and fertility behaviour among Syrian migrant women. Eur J Contracept Reprod Health Care.

[53] Amiri M, El-Mowafi IM, Chahien T (2020). An overview of the sexual and reproductive health status and service delivery among Syrian refugees in Jordan, nine years since the crisis: a systematic literature review. Reprod Health.

[54] Samari G (2017). Syrian Refugee Women’s Health in Lebanon, Turkey, and Jordan and Recommendations for Improved Practice. *World Med & Health Policy*.

[55] Al-Rousan T, Schwabkey Z, Jirmanus L (2018). Health needs and priorities of Syrian refugees in camps and urban settings in Jordan: perspectives of refugees and health care providers. East Mediterr Health J.

[56] Bakesiima R, Cleeve A, Larsson E (2020). Modern contraceptive use among female refugee adolescents in northern Uganda: prevalence and associated factors. Reprod Health.

[57] Achola R, Atuyambe L, Nabiwemba E (2024). Factors associated with family planning use among refugee and host populations in Adjumani district, West Nile, Uganda: a comparative study. BMC Public Health.

[58] World Health Organisation (2022). World report on the health of refugees and migrants.

[59] Hanschmidt F, Linde K, Hilbert A (2016). Abortion Stigma: A Systematic Review. Perspect Sex Reprod Health.

[60] Asemota OA, Klatsky P (2015). Access to infertility care in the developing world: the family promotion gap. Semin Reprod Med.

[61] Dyer SJ, Abrahams N, Mokoena NE (2005). Psychological distress among women suffering from couple infertility in South Africa: a quantitative assessment. Hum Reprod.

[62] Stellar C, Garcia-Moreno C, Temmerman M (2016). A systematic review and narrative report of the relationship between infertility, subfertility, and intimate partner violence. Int J Gynaecol Obstet.

[63] Dhont N, Muvunyi C, Luchters S (2011). HIV infection and sexual behaviour in primary and secondary infertile relationships: a case-control study in Kigali, Rwanda. Sex Transm Infect.

[64] Rouchou B (2013). Consequences of infertility in developing countries. Perspect Public Health.

[65] Rutstein S, Shah I (2004). DHS Comparative Reports 9. Infecundity, Infertility, and Childlessness in Developing Countries.

[66] Dyer SJ, Patel M (2012). The economic impact of infertility on women in developing countries ‑ a systematic review. Facts Views Vis Obgyn.

[67] Inhorn MC, Patrizio P (2015). Infertility around the globe: new thinking on gender, reproductive technologies and global movements in the 21st century. Hum Reprod Update.

[68] Yentur Doni N, Aksoy M, Simsek Z (2016). Investigation of the Prevalence of Trichomonas vaginalis Among Female Syrian Refugees with the Complaints of Vaginitis Aged Between 15-49 Years. Mikrobiyol Bul.

[69] Silverberg SL, Harding L, Spitzer RF (2018). The Who, What, Why and When of Gynaecological Referrals for Refugee Women. J Immigrant Minority Health.

[70] Balsara ZP, Wu I, Marsh DR (2010). Reproductive tract disorders among Afghan refugee women attending health clinics in Haripur, Pakistan. J Health Popul Nutr.

[71] Michelle F (2019). Social and feminist design in emergency contexts: the Women’s Social Architecture Project, Cox’s Bazar, Bangladesh. Gender \& Development.

[72] Thuita W, Conn C, Wilson K (2017). The role of marginalised women in sanitation initiatives: Somali women in northern Kenya. Dev Pract.

[73] Amnesty International (2021). Greece: violence, lies and pushbacks.

[74] European Council for Refugees and Exiles Greece: illegal pushbacks continue as arrivals drop under “strict but fair” immigration policy. https://ecre.org/greece-illegal-pushbacks-continue-as-arrivals-drop-under-strict-but-fair-immigration-policy-new-closed-controlled-camps-faces-massive-criticism.

[75] Human Rights Watch (2022). Their faces were covered” greece’s use of migrants as police auxiliaries in pushbacks.

[76] United Nations High Commissioner for Refugees UNHCR warns asylum under attack at europe’s borders, urges end to pushbacks and violence against refugees. https://www.unhcr.org/news/news-releases/unhcr-warns-asylum-under-attack-europes-borders-urges-end-pushbacks-and-violence.

[77] Turkish Coast Guard Command (2023). Pushback incidences in the year 2023. https://en.sg.gov.tr/push-back-incidents-in-the-year-of-2023.

[78] Aegean Boat Report (2023). Annual report 2023. https://aegeanboatreport.com/annual-reports.

[79] World Health Organisation (2013). Global and regional estimates of violence against women: prevalence and health effects of intimate partner violence and non-partner sexual violence: World Health Organization.

[80] Vu A, Adam A, Wirtz A (2014). The Prevalence of Sexual Violence among Female Refugees in Complex Humanitarian Emergencies: a Systematic Review and Meta-analysis. PLoS Curr.

[81] Keygnaert I, Vettenburg N, Temmerman M (2012). Hidden violence is silent rape: sexual and gender-based violence in refugees, asylum seekers and undocumented migrants in Belgium and the Netherlands. Cult Health Sex.

[82] Araujo JDO, Souza FM de, Proença R (2019). Prevalence of sexual violence among refugees: a systematic review. Rev saúde pública.

[83] Hadjicharalambous D, Parlalis S (2021). Migrants’ Sexual Violence in the Mediterranean Region: A Regional Analysis. *Sexes*.

[84] United Nations High Commissioner for Refugees (2019). Desperate journeys: refugees and migrants arriving in europe and at Europe’s borders.

[85] Belanteri RA, Hinderaker SG, Wilkinson E (2020). Sexual violence against migrants and asylum seekers. The experience of the MSF clinic on Lesvos Island, Greece. PLoS One.

[86] Araujo JDO, Souza F de, Proença R Prevalence of sexual violence among refugees: a systematic review.

