# Supplementary figures and images for: Sexual and reproductive health needs of refugee women on Lesbos, Greece: a participatory cross-sectional study
Source: BMJ Glob Health. 2026 Jun 28;11(6):e019240. doi: 10.1136/bmjgh-2025-019240 (PMC13311758; doi:10.1136/bmjgh-2025-019240)

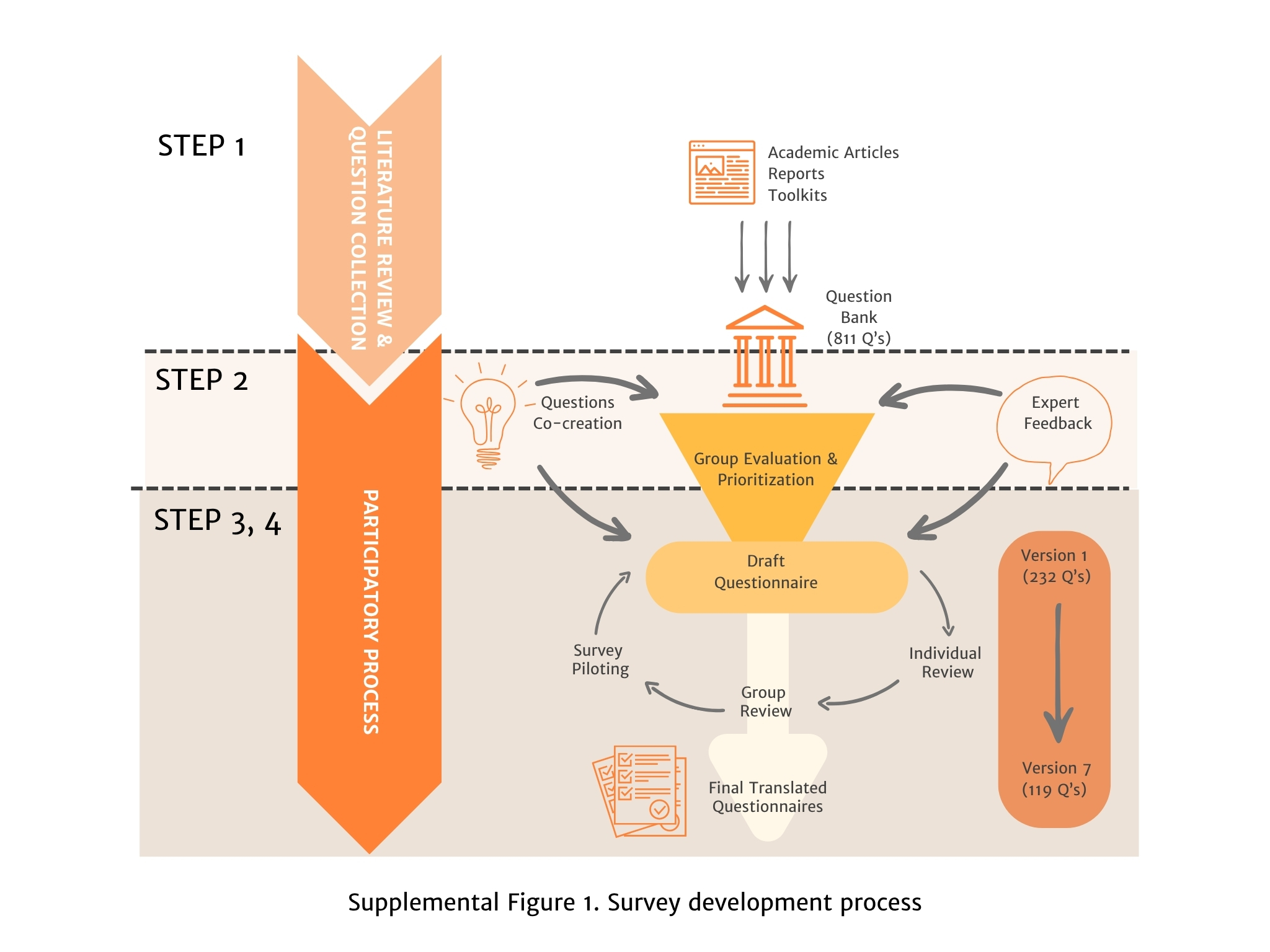

Supplement: online supplemental figure 1 [file bmjgh-11-6-s001.jpg]

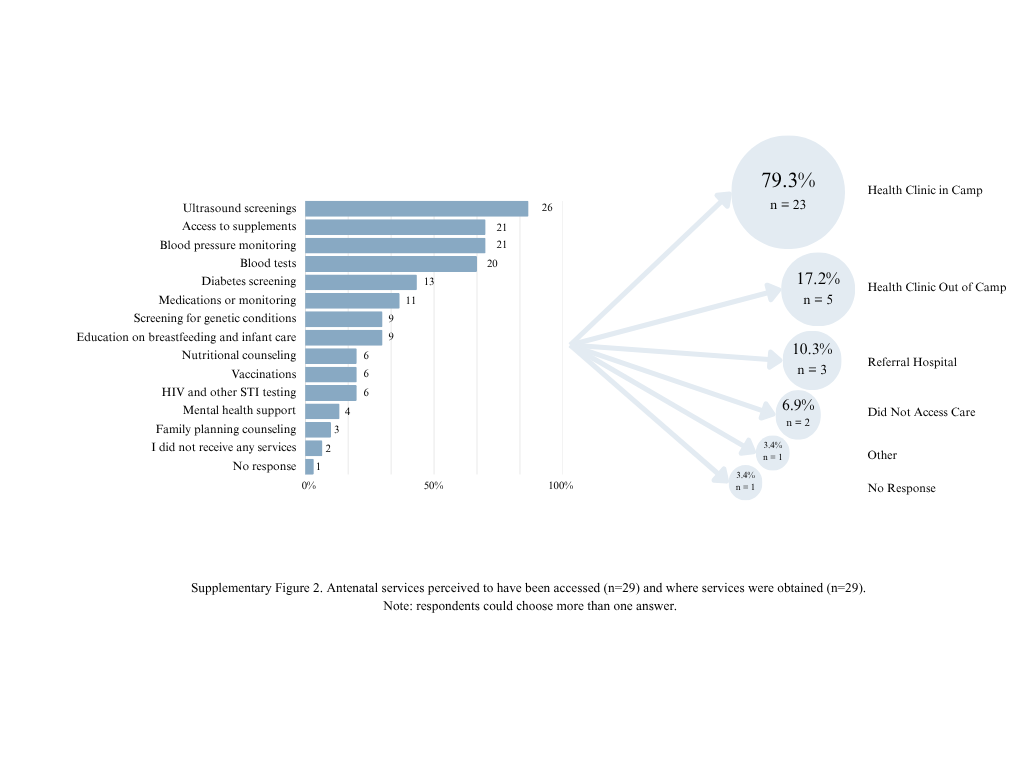

Supplement: online supplemental figure 2 [file bmjgh-11-6-s002.png]

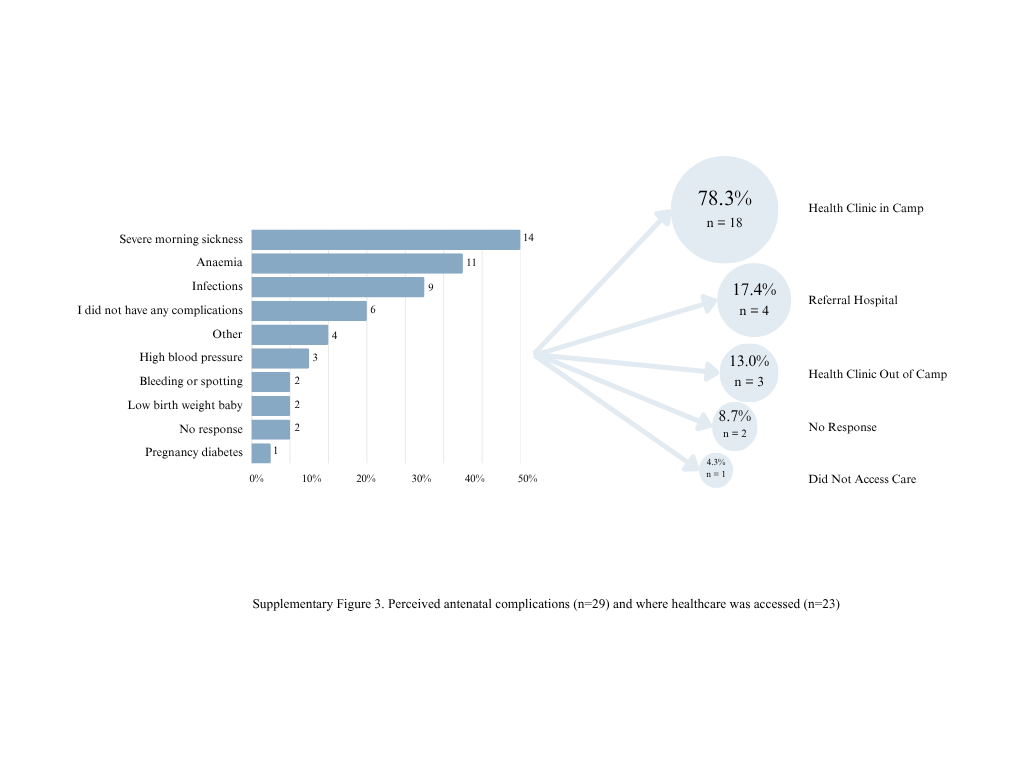

Supplement: online supplemental figure 3 [file bmjgh-11-6-s003.png]

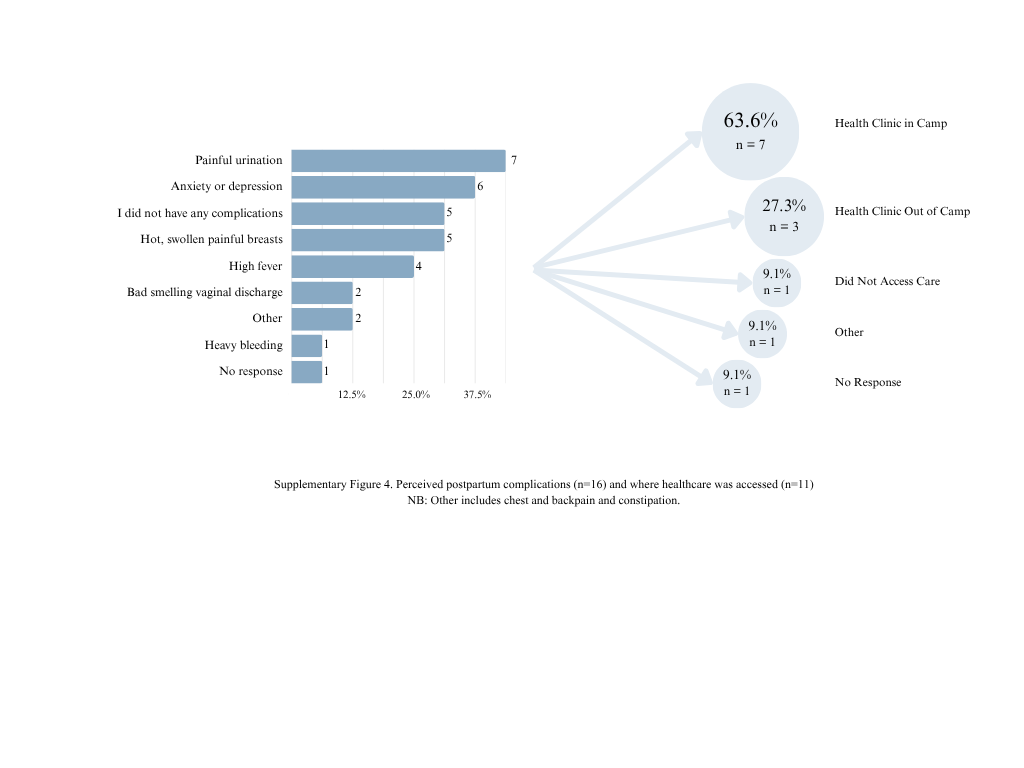

Supplement: online supplemental figure 4 [file bmjgh-11-6-s004.png]

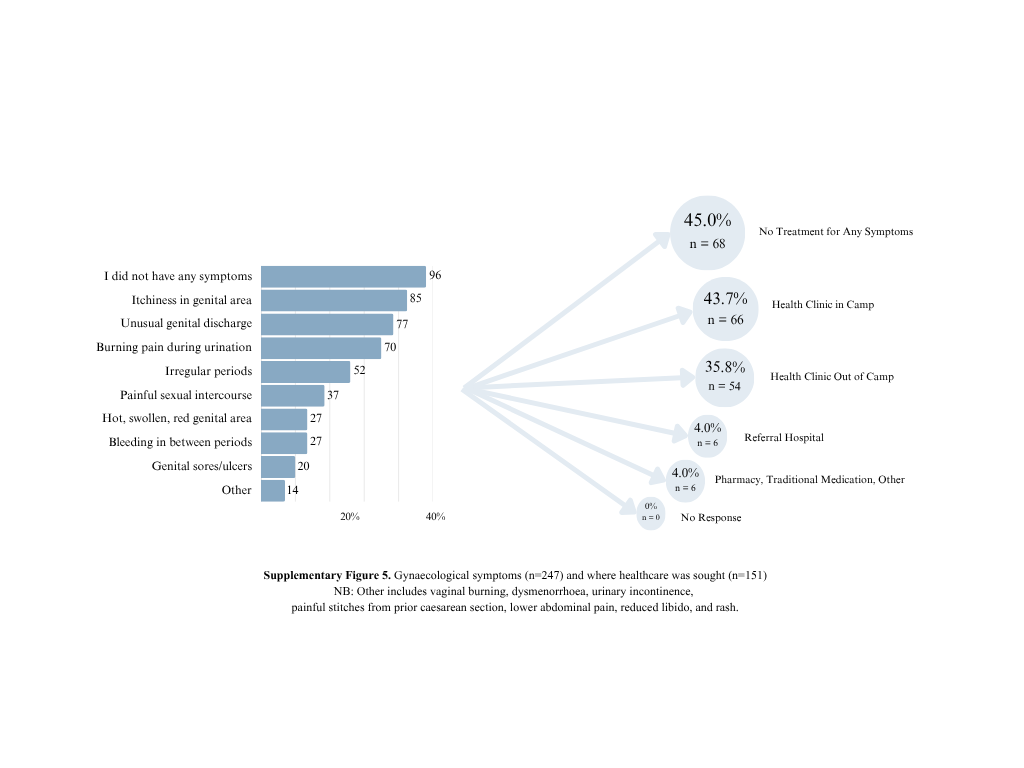

Supplement: online supplemental figure 5 [file bmjgh-11-6-s005.png]

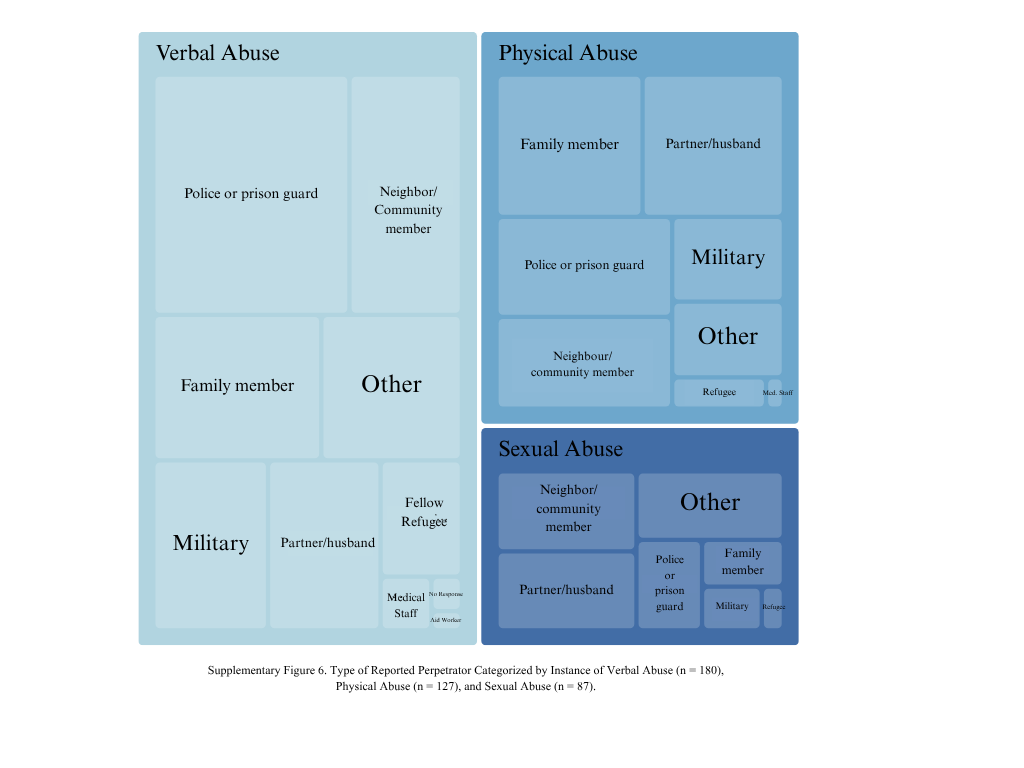

Supplement: online supplemental figure 6 [file bmjgh-11-6-s006.png]

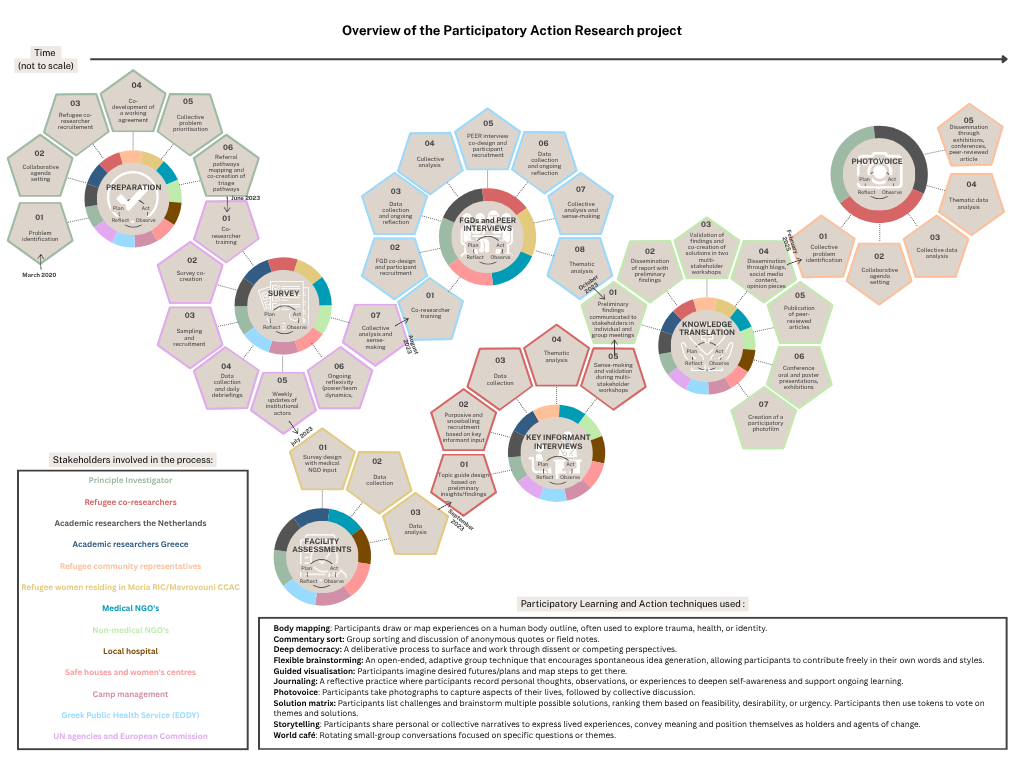

Supplement: online supplemental file 1 [file bmjgh-11-6-s007.png]
