# Supplementary material for: Sexual and reproductive health needs of refugee women on Lesbos, Greece: a participatory cross-sectional study
Source: BMJ Glob Health. 2026 Jun 28;11(6):e019240. doi: 10.1136/bmjgh-2025-019240 (PMC13311758; doi:10.1136/bmjgh-2025-019240)
Supplement: online supplemental file 2 [file bmjgh-11-6-s008.docx]

AUTHOR REFLEXIVITY STATEMENT

1. **How does this study address local research and policy priorities?**

This study originated in 2020, when JS (the principal investigator and medical coordinator of an NGO at the time) and HJ (a medical translator and later co-researcher) worked closely together in a medical post on Lesbos and encountered numerous unmet sexual and reproductive health (SRH) needs among refugee women. These shared experiences sparked ongoing conversations and a commitment to address this gap in a more systematic and participatory way. JS and HJ remained in close contact after JS returned to the Netherlands, and between 2020 and 2023, JS made several return visits to Lesbos to deepen collaboration and develop the research approach.

During this time, JS established partnerships with AT and EK (affiliated with the National and Kapodistrian University of Athens) and SS (then head of the National Public Health Organisation, EODY), as well as with other key stakeholders, including camp management, various (medical) camp actors, refugee residents and community representatives. As a result, the study (design) reflects locally identified needs and policy gaps, with SRH selected as a priority area based on ongoing dialogue with affected communities.

1. **How were local researchers involved in study design?**

Local researchers were closely involved in shaping the study from the outset. During the preparatory phase, JS conducted regular consultations with HJ (who later became one of the co-researchers) and other community members to ensure the study reflected lived realities and locally relevant concerns. Once in the field, the academic team and co-researchers co-developed key aspects of the study design, including refining research questions, developing the study tool, and determining appropriate recruitment strategies. Training workshops also created space for critical input on ethical considerations and logistical feasibility. This collaborative process ensured the study was not externally imposed but co-created with those embedded in the setting.

1. **How has funding been used to support the local research team?**

Each co-researcher (ZA, LBB, EB, ZH, HJ, MJ, KM, SM, FM) received a monthly salary throughout the research period. Two master’s students (AB, CC) each received a fixed stipend to cover flights and accommodation. Daily transport and lunch were provided for the entire team to support their participation. Funding also covered shared resources, including laptops, mobile phones, and Wi-Fi, ensuring the team could work independently and flexibly. Additionally, co-researchers were included in dissemination activities, and funds have been allocated to support their ongoing involvement in future outputs (such as conference fees) and advocacy efforts. The Greek academic partners, led by EK and AT, received a financial contribution to support their institutional and advisory assistance.

1. **How are research staff who conducted data collection acknowledged?**

All are included as authors on this paper and explicitly mentioned in any dissemination products.

1. **Do all members of the research partnership have access to study data?**

Yes, all members of the research partnership have access to the study data.

1. **How was data used to develop analytical skills within the partnership?**

Analytical skill-building was integrated into the participatory process from the outset. Co-researchers were trained in qualitative and quantitative methods, including how to interpret descriptive statistics and identify patterns within the data. During a full-day sense-making session, the team collaboratively reviewed preliminary findings (sometimes presented visually through graphs and charts) and discussed whether these resonated with their lived experiences. This process enabled co-researchers to critically assess the data, clarify inconsistencies, and offer contextual explanations, thereby deepening collective analytical insight. The collaborative interpretation of findings fostered a shared understanding of the results and strengthened the team’s capacity for future research engagement.

1. **How have research partners collaborated in interpreting study data?**

Data interpretation was a collaborative effort involving co-researchers and academic researchers. Additionally, findings were presented in two participatory workshops with policy, academic, and NGO stakeholders, where feedback informed the framing of results and helped identify priority areas for action.

1. **How were research partners supported to develop writing skills?**

Co-researchers were invited to contribute to the writing process through reviewing manuscript drafts for which they often provided verbal feedback.

1. **How will research products be shared to address local needs?**

Research findings will be shared through multiple formats tailored to different audiences. Findings have already been presented to local health actors, non-medical NGOs, camp authorities and international UN agencies in a co-organised session on Lesbos, with the aim of informing service delivery. In addition, two co-creation workshops were held with policymakers, practitioners, academics, and refugee women to validate the findings, prioritise action areas, and collaboratively develop context-appropriate solutions.

1. **How is the leadership, contribution and ownership of this work by LMIC researchers recognised within the authorship?**

Leadership, contribution, and ownership by researchers from LMICs are reflected throughout the research process and authorship structure. The co-researchers’ involvement is recognised throughout the participatory approach and reflected through co-authorship. In addition, Greek academic collaborators (SS, EK, AT) who played a central role in local coordination, stakeholder engagement, and supervision, are included as co-authors. This approach reflects our commitment to equitable authorship that acknowledges both intellectual and practical contributions across contexts.

1. **How have early career researchers across the partnership been included within the authorship team?**

AB and CC, master’s students at the time, are listed as second and third authors on the manuscript, reflecting their level of involvement in the design of data collection tools, data collection and curation, analysis, and contributions to manuscript drafts.

1. **How has gender balance been addressed within the authorship?**

JS, AB, CC, ZA, LB, EB, ZH, HJ, MJ, KM, SM, EK, MLM, and MvdM identify as women, while FM, SS, AT, NY, and TvdA identify as men. Given that our study population consisted of women, we intentionally structured the research team to reflect this reality; not only by considering gender identity, but also through an intersectional lens. Notably, many of the women co-authors share lived experiences of displacement with the study population.

Recognising that SRH is often framed narrowly as a "women’s issue," and following explicit advice from the Congolese community representatives, we purposively included one male co-researcher. We believe that achieving gender equity in SRH requires engaging men not only as participants but also as collaborators. In this sense, our approach sought to balance feminist research principles with inclusive and community-informed praxis.

1. **How has the project contributed to training of LMIC researchers?**

The co-researcher team participated in a 3-week training programme covering research methodologies, foundational knowledge of each SRH domain, ethical considerations (informed consent, confidentiality), positionality, trauma-informed and non-judgemental interviewing techniques, psychological first aid, de-escalation strategies, computer skills, triage and referral pathways. The training was designed to equip co-researchers with both technical skills and critical reflection tools necessary for rigorous data collection and analysis.

Beyond the initial training, ongoing mentorship and support were provided throughout the entire research process. This included daily debriefings during fieldwork, collaborative data interpretation sessions, individual check-ins, CV-development workshops and active involvement in manuscript development and dissemination activities.

Engagement with the academic research team has continued beyond data collection, fostering sustained skill development and research leadership. Co-researchers have presented study findings at inter-stakeholder workshops and in-house symposia and are currently preparing for poster and oral presentations at an international conference. Individual mentoring on career development, study opportunities, and reference letter support is also provided as needed.

This approach ensured that capacity strengthening was not limited to a one-time event but evolved into a dynamic, ongoing partnership, empowering co-researchers to take ownership of the research.

1. **How has the project contributed to improvements in local infrastructure?**

The project has contributed to fostering greater inter-agency collaboration in the field of SRH on Lesbos by bringing together a range of actors through joint workshops, co-creation sessions, and informal exchanges. The project created space for more coordinated dialogue and mutual learning, bridging the gap between academic research and humanitarian fieldwork.

1. **What safeguarding procedures were used to protect local study participants and researchers?**

Safeguarding was a core consideration throughout the study. All co-researchers received training in research ethics, trauma-informed care, and psychological first aid, and were instructed on how to handle disclosures of distress or harm. Interviewers used a non-judgemental approach, and interviews were conducted in safe, private spaces chosen by the respondent. Respondents were informed that participation was voluntary and that they could withdraw at any time without consequence.

To protect the well-being of the co-researchers, we implemented daily debriefings, individual check-ins, group mental health sessions and peer support mechanisms. A referral system was established for both respondents and co-researchers in need of psychosocial support. Confidentiality was strictly maintained throughout data handling, and identifying information was never recorded.
