# Supplementary material for: Sexual and reproductive health needs of refugee women on Lesbos, Greece: a participatory cross-sectional study
Source: BMJ Glob Health. 2026 Jun 28;11(6):e019240. doi: 10.1136/bmjgh-2025-019240 (PMC13311758; doi:10.1136/bmjgh-2025-019240)
Supplement: online supplemental file 3 [file bmjgh-11-6-s009.pdf]

Survey questions and answers

Note that for the questions marked with an asterix (*) respondents were able to provide more than one answer. Therefore, percentages may not always total 100%.

Question/ Responses (n, missing)	Value	Percentage (%)
**GENERAL INFORMED CONSENT**		
Hi, my name is (your name), I am a refugee from (your country). I am part of a research group from the University of Amsterdam and the University of Athens. We are researching women's health in this camp. I would like to ask you some questions about your health (for example about pregnancy and contraception). I cannot promise to help your situation, but with the information you give us, we hope to improve the services in camp in the future. I am not a medical professional but if a health condition comes up, I can refer you to a service provider in camp. Everything you tell me stays between us and will not be shared with other refugees, NGOS's, and the government. This research will not have an effect on your asylum procedure. Your name and contact details will be stored safely and only be shared (with our coordinator Jamilah) in case of an emergency for your health. Some of our questions are sensitive. If at any point you don't want to answer a question, or you want to stop the interview, you can do so without any consequences. If at any point you want me to delete your information, that is also possible. This interview will take approximately 60 minutes. Do you have any questions? Do you assent/consent to participate?		
**DEMOGRAPHICS AND DISPLACEMENT CHARACTERISTICS**		
**How old are you? (n = 247, m = 0)**	24 (IQR 21-29.5)	100
**Which country are you from? (n = 247, m = 0)**		
Afghanistan	86	34.8
Eritrea	67	27.1
Somalia	30	12.1
Yemen	17	6.9
Palestine	13	5.3
Democratic Republic of Congo	12	4.9
Syria	6	2.4
Sierra Leone	5	2.0
Other (Sudan, Ethiopia, Cameroon, Iraq, Uganda, Iran, Guinea, Liberia, Tajikistan)	11	4.5
No response	0	0.0
**Which religion do you identify with? (n = 247, m = 0)**		
Muslim	183	74.1
Christian	61	24.7
Other (Kimbangiusm)	3	1.2
No response	0	0.0
**What is the highest level of education you completed? (n = 247, m = 0)**		
Never attended school	73	29.6
Primary	76	30.8
Secondary	44	17.8
Post-secondary	51	20.6
Other	3	1.2
No response	0	0.0
**Are you part of the LGBTQI community? (n = 246, m = 1)**		
Yes	1	0.4
No	245	99.2
No response	0	0.0
**Are you currently living with a partner or husband? (n = 247, m = 0)**		
Yes	90	36.4
No	157	63.6
No response	0	0.0
**In what year did you first leave your home? (n = 246, m = 1)**		
Median Years Displaced (n = 246)	2 (IQR 1–5, range 0-32)	100
No response	0	0.0
**How many months have you been living here in camp? (n = 247, m = 0)**		

Median Months in Camp (n = 247)	4 (IQR 2–7, range 0-58)	100
No response	0	0
**Did you experience any pushbacks before arriving? (n = 247, m = 0)**		
Yes	133	53.8
No	111	44.9
No response	3	1.2
**How many pushbacks did you experience? (n = 133, m = 0)**		
Median Number Pushbacks (n = 132)	2 (IQR 1-4, range 1-42)	99.2
No Response	1	0.8
**Have you received a negative response to your request for asylum? (n = 247, m = 0)**		
Yes	69	27.9
No	178	72.1
No response	0	0
**How often have you been denied medical care because of your legal status? (n = 247, m = 0)**		
Always	8	3.2
Often	14	5.7
Sometimes	24	9.7
Rarely	11	4.5
Never	187	75.7
No response	3	1.2
**MATERNAL HEALTH**		
**I now have some questions about pregnancy and motherhood. Are you comfortable continuing? (n = 247)**		
Yes	240	97.2
No	7	2.8
**Where do you go to get information about issues related to care before, during and after pregnancy? (n = 239, m = 1)***		
I did not get information	170	71.1
Healthcare professionals	39	16.3
Trained volunteers or counselors from NGOs	29	12.1
Community leaders or representatives	0	0.0
Religious or spiritual leaders	0	0.0
Female family members or trusted female friends	7	2.9
Pamphlets or educational materials provided in the camp	0	0.0
Online resources or websites	3	1.3
Other (word-of-mouth)	1	0.4
No response	2	0.8
**Where do you prefer to get information about issues related to care before, during and after pregnancy? (n = 239, m = 1)***		
I prefer not to receive information	43	18.0
Healthcare professionals	163	68.2
Trained volunteers or counselors from NGOs	35	14.6
Community leaders or representatives	2	0.8
Religious or spiritual leaders	0	0.0
Female family members or trusted female friends	9	3.8
Pamphlets or educational materials provided in the camp	5	2.1
Online resources or websites	15	6.3
Other (word-of-mouth (2), books (1), life experience (1), no preference (1))	5	2.1
No response	3	1.3
**Are you currently pregnant? (n = 240, m = 0)**		
Yes	18	7.5
No	221	92.1
No response	1	0.4
**How long have you been pregnant? (n = 18, m = 0)**		
Mean Number of Months (n = 18)	4.24 (IQR 2–6)	100
No response	0	0.0
**How many children do you have? (n = 239, m = 1)** *NOTE: 131 women had children. The 108 women who did not have any children were included in the “Median Number of Children” calculation.*		

Median Number of Children (n = 239)	1.0 (IQR 0-3, range 0-13)	100
No response	0	0.0
**Were any of your children born during your time in this camp, did you arrive in camp with a baby under 6 weeks old or are you currently breastfeeding?* (n = 124, m = 7)** *NOTE: 4 women selected both "Yes, born in camp" and "Yes, I am currently breastfeeding".*		
Yes, born in camp	16	12.9
Yes, arrived with a baby under 6 weeks old	0	0.0
Yes, I am currently breastfeeding	12	9.7
No	100	80.6
No response	0	0.0
Antenatal Care (During your last or current pregnancy…):		
**How many months pregnant were you when you had your first visit for antenatal care? (n = 28, m = 6)** *NOTE: all women who were pregnant and all women who had children born in camp were asked this question. No women who had given birth in camp previously, were also pregnant at the time of survey.*		
I did not go for care before or during pregnancy	2	7.1
Median number of months (n = 25)	4.0 (IQR 1.8-7.0, range 0.8-9)	89.3
No response	1	3.6
**How many times did you see someone for antenatal care? (n = 28, m = 6)**		
Number of Times (n = 27)	7 (IQR 2-10, range 0-28)	96.4
No response	1	3.6
**Before or during your pregnancy, did you receive any of the following services? (n = 29, m = 5)***		
I did not receive any of the following services	2	6.9
Ultrasound screenings	26	89.7
Screening for genetic conditions in your unborn baby	9	31.0
Blood tests, for example for anaemia	20	69.0
Nutritional counselling	6	20.7
Access to supplements such as folic acid and multivitamins	21	72.4
Vaccinations	6	20.7
HIV and other STI testing	6	20.7
Blood pressure monitoring	21	72.4
Diabetes screening	13	44.8
Mental health support	4	13.8
Education on breastfeeding and infant care	9	31.0
Medications or monitoring for problems during pregnancy	11	37.9
Family planning counselling	3	10.3
Other	0	0.0
No response	1	3.4
**Where did you go for your antenatal care visits? (n = 29, m = 5)***		
I did not seek care	2	6.9
Health clinic in camp (BRF, CMA, EODY, MDM)	23	79.3
Health clinic out of camp (MSF)	5	17.2
Referral hospital	3	10.3
Other (NGO)	1	3.4
No response	1	3.4
**During your pregnancy or labour, did you have any of the following complications? (n = 29, m = 5)***		
I did not have any complications	6	20.7
Severe morning sickness	14	48.3
Pregnancy diabetes	1	3.4
High blood pressure	3	10.3
Anaemia	11	37.9
Infections (such as urinary tract infections or vaginal infections)	9	31.0
Bleeding or spotting	2	6.9
Preterm labour or premature birth	0	0.0
Low birth weight baby	2	6.9
Problems with the placenta	0	0.0

Multiple pregnancies (twins, triplets, etc.)	0	0.0
Foetal growth restriction	0	0.0
Other (Back & Chest Pain (1), Fibroids & Breech Presentation (1), Low Blood Pressure & Fatigue (1), Uterine Rupture (1))	4	13.8
No response	2	6.9
**Where did you go to get help for the complications associated with your pregnancy or labour? (n = 23, m = 0)***		
I did not seek care	1	4.3
Health clinic in camp (BRF, CMA, EODY, MDM)	18	78.3
Health clinic out of camp (MSF)	3	13.0
Referral hospital	4	17.4
Other	0	0.0
No response	2	8.7
**During your pregnancy, how often did you receive food that was different from normal? (n = 29, m = 5)**		
Always	2	6.9
Often	3	10.3
Sometimes	9	31.0
Rarely	1	3.4
Never	12	41.4
No response	2	6.9
**During your pregnancy, how often did you receive MORE food than normal? (n = 29, m = 5)**		
Always	2	6.9
Often	0	0.0
Sometimes	5	17.2
Rarely	1	3.4
Never	19	65.5
No response	2	6.9
Perinatal care		
**Where was your last child born? (n = 15, m = 1)**		
In my home in camp	0	0.0
In a clinic in camp	0	0.0
In a hospital outside camp	14	93.3
Other	0	0.0
No response	1	6.7
**How was your last child born? (n = 15, m = 1)**		
Vaginally	9	60.0
Caesarean (C-section)	5	33.3
No response	1	6.7
**Which statement best describes whose idea it was for you to have a c-section? (n = 5, m = 0)**		
My health care provider recommended a caesarean delivery before I went into labour (i.e.: primary C-section)	3	60.0
My health care provider recommended a caesarean delivery while I was in labour (i.e.: secondary C-section)	2	40.0
I asked for the caesarean delivery	0	0.0
I don't know	0	0.0
No response	0	0.0
**If a doctor, nurse, or other health care worker tried to induce your labour (start your contractions using medicine), why did they do so? (n = 15, m = 1)**		
My labour was not induced	8	53.3
My water broke and there was a fear of infection	0	0.0
I was past my due date	1	6.7
My health care provider worried about the size of the baby	0	0.0
My baby was not doing well and needed to be born	1	6.7
I had a complication in my pregnancy (such as high blood pressure)	1	6.7
I wanted to schedule my delivery	0	0.0
I don't know	1	6.7
Other (maternal fatigue (1), myomas (1))	2	13.3

No response	1	6.7
**Since you have been living inside the camp, how many children did you have who were born dead BEFORE completing 6 months of pregnancy? (n = 238, m = 2)**		
None	235	98.7
Median Number Miscarriages (n = 2)	1 (range 1 – 1)	0.8
No response	1	0.4
**Since you have been living inside the camp, how many children did you have who were born dead AFTER completing 6 months of pregnancy? (n = 237, m = 3)**		
None	236	99.6
Median Number Stillbirths (n = 0)	0	0.0
No response	1	0.4
**Since you have been living inside the camp, how many children did you have who were born alive but died within ONE HOUR after birth? (n = 238, m = 2)**		
None	237	99.6
Median Number Perinatal Deaths (n = 0)	0	0.0
No response	1	0.4
**Since you have been living inside the camp, how many children did you have who were born alive and died before ONE MONTH after birth? (n = 238, m = 2)**		
None	237	99.6
Median Number Neonatal Deaths (n = 0)	0	0.0
No response	1	0.4
**During your journey by land/ sea to this camp in Greece, how many of your children under the age of 18 died? (n = 238, m = 2)**		
None	235	98.7
Median number deaths at sea (n = 2)	1 (range 1 – 1)	0.8
No response	1	0.4
Post-partum care		
**During the 6 weeks after birth, how many times did you go to the health centre to check your health? (n = 15, m = 1)** *NOTE: 7 women who selected "I did not go to the health centre" were included in the "Median Number of Health Centre Visits" calculation.*		
I did not go to the health centre	7	46.7
Median Number of Health Centre Visits (n = 14)	0.5 (IQR 0.0-4.3, range 0-10)	93.3
No response	1	6.7
**During the 6 weeks after birth, did you have any of the following problems or complications: (n = 16, m = 0)***		
I did not have any complications	5	31.3
Heavy bleeding	1	6.3
Bad smelling vaginal discharge	2	12.5
High fever	4	25.0
Painful urination	7	43.8
Hot, swollen painful breasts	5	31.3
Anxiety or depression	6	37.5
Other (chest and back pain (1), constipation (1))	2	12.5
No response	1	6.3
**Where did you seek help for these problem(s) or complication(s)? (n = 11, m = 0)***		
I did not seek care	1	9.1
Health clinic in camp (BRF, CMA, EODY, MDM)	7	63.6
Health clinic out of camp (MSF)	3	27.3
Referral hospital	0	0.0
Other (over the counter medication)	1	9.1
No response	1	9.1
Breastfeeding		
**Where do you go to get information about issues related to breastfeeding? (n = 22, m = 2)***		
I did not get information	10	45.5
Healthcare professionals	6	27.3
Trained volunteers or counselors from NGOs	7	31.8
Community leaders or representatives	0	0.0

Religious or spiritual leaders	0	0.0
Female family members or trusted female friends	1	4.5
Pamphlets or educational materials provided in the camp	0	0.0
Online resources or websites	0	0.0
Other	0	0.0
No response	1	4.5
**Where do you prefer to go to get information about issues related to breastfeeding? (n = 22, m = 2)***		
I prefer not to receive information	2	9.1
Healthcare professionals	12	54.5
Trained volunteers or counsellors from NGOs	9	40.9
Community leaders or representatives	0	0.0
Religious or spiritual leaders	0	0,0
Female family members or trusted female friends	0	0,0
Pamphlets or educational materials provided in the camp	0	0,0
Online resources or websites	2	9.1
Other (life experience (1), no preference (1))	2	9.1
No response	1	4.5
**How many weeks or months did you breastfeed or feed pumped milk to your baby? (n = 22, m = 2)** *NOTE: "Median months baby was breastfed" calculation excluded the women who were still breastfeeding.*		
I did not breastfeed or feed pumped milk to my baby	0	0.0
Median months baby was breastfed (n = 10)	3.0 (IQR 1.6-5.8, range 0.5-11)	45.5
I am currently still breastfeeding	11	50.0
No response	1	4.5
**How old was your new baby the first time he or she had liquids other than breast milk (such as formula, water, juice or cow's milk)? (n = 22, m = 2)**		
My baby has not had any liquids other than breast milk	5	22.7
Mean Age in Months (n = 14)	3.7 (IQR 1.4-5.8, range 0.3-8.0)	63.6
No response	3	13.6
**How old was your new baby the first time he or she ate food (such as baby cereal, baby food, or any other food)? (n = 22, m = 2)**		
My baby has not had any food yet	10	45.5
Median Age in Months (n = 10)	4.5 (IQR 2.3-6.0, range 1.0-7.0)	45.5
No response	2	9.1
**FAMILY PLANNING**		
**Family planning has to do with how many children you choose to have and when. It includes methods used to prevent having children as well as methods to try to have children. I would now like to ask you some questions about this. Are you comfortable continuing? (n = 247, m = 0)**		
Yes	242	98.0
No	5	2.0
**Where do you currently go to get information about issues related to family planning and contraception? (n = 242, m = 0)***		
I did not get information	163	67.4
Healthcare professionals	49	20.2
Trained volunteers or counselors from NGOs	35	14.5
Community leaders or representatives	0	0.0
Religious or spiritual leaders	0	0.0
Female family members or trusted female friends	7	2.9
Pamphlets or educational materials provided in the camp	0	0.0
Online resources or websites	5	2.1
Other (word-of-mouth)	1	0.4
No response	0	0.0
**Where do you prefer to get information about issues related to family planning and contraception? (n = 242, m = 0)***		
I prefer not to receive information	40	16.5
Healthcare professionals	173	71.5
Trained volunteers or counselors from NGOs	43	17.8

Community leaders or representatives	3	1.2
Religious or spiritual leaders	2	0.8
Female family members or trusted female friends	8	3.3
Pamphlets or educational materials provided in the camp	3	1.2
Online resources or websites	17	7.0
Other	1	0.4
No response	0	0.0
**Do you want to prevent having a baby? (n = 242, m = 0)**		
Yes	176	72.7
No	63	26.0
No response	3	1.2
**Are you using any of the following methods to prevent having a baby? (n = 178, m = 1**)* *NOTE: All of the women who answered 'no response' to 'do you want to prevent having a baby?', were asked/answered this question.*		
I am not using any methods	45	25.3
Abstinence	91	51.1
Male condoms	22	12.4
Withdrawal	18	10.1
IUD	9	5.1
Oral contraceptives	6	3.4
Rhythm/calendar method	5	2.8
Injectables	4	2.2
Tubal ligation	1	0.6
Female condoms	0	0.0
Implants	0	0.0
Emergency hormonal contraception	0	0.0
Other	0	0.0
No response	3	1.7
**Where do you go to get your method to prevent having a baby? (n = 130, m = 4)***		
Using Abstinence, Rhythm/Calendar Method or Withdrawal	84	64.6
Health clinic out of camp	25	19.2
Health clinic in camp	4	3.1
I got it before arriving in camp	4	3.1
Pharmacy	2	1.5
Supermarket	1	0.8
Other (NGO's)	8	6.2
No response	6	4.6
**What is your preferred method to prevent you from having a baby? (n = 242, m = 0)***		
I do not have a preference	61	25.2
Male condoms	35	14.5
Oral contraceptives	30	12.4
Withdrawal	28	11.6
IUD	28	11.6
Abstinence	24	9.9
Implants	22	9.1
Injectables	17	7.0
Tubal ligation	8	3.3
Rhythm/Calendar Method	4	1.7
Female condoms	1	0.4
Emergency hormonal contraception	1	0.4
No response	2	0.8
**How many times have you used emergency contraception/morning-after pill? (n = 242, m = 0)**		
I did not use emergency contraception/morning after pill	230	95.0
Mean Number Times (n = 12)	5.17 (IQR 1.0-2.5, range 1-24)	5.0
No response	0	0.0
**Are you currently using any treatment to get pregnant? (method to try having a baby if you are unable to)? (n = 66, m = 0)**		

Yes, fertility-enhancing drugs	0	0.0
Yes, artificial insemination	0	0.0
Yes, assisted reproductive technology	0	0.0
Yes, traditional methods	0	0.0
Yes, other	0	0.0
No, and I do not want to	48	72.7
No, but I want to	17	25.8
No response	1	1.5
**SEXUALLY TRANSMITTED INFECTIONS (STIS)**		
**Sexually transmitted infections (STIs) are infections that are spread mostly by unsafe sexual contact (i.e.: without a condom). Some STIs can also be transmitted during pregnancy, childbirth and breastfeeding and through infected blood. With your permission, I am going to ask you some questions about this topic. Are you feeling comfortable with continuing? (n = 247, m = 0)**		
Yes	240	97.2
No	7	2.8
**Where do you currently go to get information about issues related to STIs and HIV? (n = 240, m = 0)***		
I did not get information	186	77.5
Healthcare professionals	33	13.8
Trained volunteers or counselors from NGOs	22	9.2
Community leaders or representatives	0	0.0
Religious or spiritual leaders	1	0.4
Female family members or trusted female friends	4	1.7
Pamphlets or educational materials provided in the camp	0	0.0
Online resources or websites	7	2.9
Other (books (1), husband (1), life experience (1), school (1))	4	1.7
No response	0	0.0
**Where do you prefer to get information about issues related to STIs and HIV? (n = 240, m = 0)***		
I prefer not to receive information	34	14.2
Healthcare professionals	171	71.3
Trained volunteers or counselors from NGOs	48	20.0
Community leaders or representatives	3	1.3
Religious or spiritual leaders	2	0.8
Female family members or trusted female friends	7	2.9
Pamphlets or educational materials provided in the camp	3	1.3
Online resources or websites	22	9.2
Other (books (1), television (1), no preference (2))	4	1.7
No response	0	0.0
**Have you had a STI/HIV test since arriving at camp? (n = 240, m = 0)**		
Yes, and I got the result	40	16.7
Yes, but I did not get the result	3	1.3
No	197	82.1
No response	0	0.0
**Where did you go to get tested? (n = 43, m = 0)***		
Health clinic in camp (BRF, CMA, MDM, EODY)	9	20.9
Health clinic out of camp (MSF)	32	74.4
Referral hospital	6	14.0
Other	0	0.0
No response	0	0.0
**MENSTRUAL AND GYNAECOLOGICAL HEALTH**		
**Now I would like to ask you some questions about your periods. Are you comfortable continuing? (n = 247, m = 0)**		
Yes	247	100.0
No	0	0.0
**Where do you currently go to get information about issues related to menstrual health?* (n = 247, m = 0)**		
I did not get information	201	81.4
Healthcare professionals	25	10.1
Trained volunteers or counselors from NGOs	17	6.9

Community leaders or representatives	0	0.0
Religious or spiritual leaders	0	0.0
Female family members or trusted female friends	7	2.8
Pamphlets or educational materials provided in the camp	0	0.0
Online resources or websites	5	2.0
Other (books (2))	2	0.8
No response	0	0.0
**Where do you prefer to get information about issues related to menstrual health?* (n = 247, m = 0)**		
I prefer not to receive information	26	10.5
Healthcare professionals	191	77.3
Trained volunteers or counselors from NGOs	51	20.6
Community leaders or representatives	2	0.8
Religious or spiritual leaders	2	0.8
Female family members or trusted female friends	8	3.2
Pamphlets or educational materials provided in the camp	8	3.2
Online resources or websites	25	10.1
Other (books (1), pharmacy (1))	2	0.8
No response	0	0.0
**Have you had a menstrual period since arriving in camp? (n =247, m = 0)**		
Yes, I have had a menstrual period since arriving in camp	207	83.8
No, I have not had a menstrual period since arriving in camp	22	8.9
I am currently pregnant and have not had a menstrual period	5	2.0
I am currently breastfeeding and have not had a menstrual period	11	4.5
I am going through menopause and have not had a menstrual period	0	0.0
I have a medical condition that affects my menstrual cycle	2	0.8
Other	0	0.0
No response	0	0.0
**During your last menstrual period, did you have enough menstrual materials to change them as often as you wanted to throughout your menstrual period?* (n = 207, m = 0)**		
I had enough menstrual materials to change them as often as I wanted to throughout my period	173	83.6
I did not have enough menstrual materials to change them as often as I wanted to throughout my period	28	13.5
I had to rely on improvised or inadequate materials during my period	11	5.3
I had to borrow menstrual materials from others during my period	4	1.9
I had to use the same menstrual materials for longer than recommended during my period	3	1.4
Other	0	0.0
No response	0	0.0
**During your last menstrual period, were you able to reduce your pain when you needed to? (n = 207, m = 0)**		
I did not have pain	53	25.6
Always	29	14.0
Often	36	17.4
Sometimes	34	16.4
Rarely	41	19.8
Never	14	6.8
No response	0	0.0
**How did you reduce your period pain? (n = 154, m = 0)***		
I used home remedies or self-care techniques to reduce the pain	94	61.0
I bought medication to reduce the pain	28	18.2
I went to the doctor to help to reduce the pain	40	26.0
Other (nothing)	23	14.9
No response	0	0.0
**During your last menstrual period, what materials did you MAINLY use to absorb or catch menstrual blood?* (n = 207, m = 0)**		
Single use menstrual pads/liners	191	92.3
Reusable menstrual pads	1	0.5

Tampons	11	5.3
Menstrual cup	0	0.0
Cloth or cotton wool	1	0.5
Toilet paper	0	0.0
Underwear only (non-absorbent)	0	0.0
Nothing	1	0.5
Other (diapers (5))	5	2.4
No response	1	0.5
**Of all the different menstrual material options, which ones would you PREFER if you could choose anything you wanted?* (n = 246, m = 1)**		
I do not have a preference	7	2.8
Single use menstrual pads/liners	213	86.6
Reusable menstrual pads	0	0.0
Tampons	18	7.3
Menstrual cup	1	0.4
Cloth or cotton wool	2	0.8
Toilet paper	2	0.8
Underwear only (non-absorbent)	0	0.0
Nothing	0	0.0
Other (diapers (10))	10	4.1
No response	3	1.2
**During your last menstrual period, was the place you most often changed your menstrual materials in camp:* (n = 205, m = 2)**		
Clean	126	61.5
Private (you didn't worry you could be seen)	168	82.0
Safe (you didn't worry you could be harmed)	157	76.6
With water	197	96.1
With soap	19	9.3
I was not using the toilet in camp during last menstrual period	0	0.0
No response	1	0.5
**Have you received a screening for cervical cancer since living in the camp? (n = 246, m = 1)**		
Yes	1	0.4
No	245	99.6
No response	0	0.0
**Where did you receive screening for cervical cancer? (n = 1, m = 0)**		
Health clinic in camp (BRF, CMA, MDM, EODY)	1	100.0
Health clinic out of camp (MSF)	0	0.0
Referral hospital	0	0.0
Other	0	0.0
No response	0	0.0
**Since arriving in camp, have you had any of the following symptoms?* (n = 247, m = 0)***		
I did not have any symptoms	96	38.9
Genital sores/ulcers	20	8.1
Unusual genital discharge	77	31.2
Hot, swollen, red genital area	27	10.9
Bleeding in between periods	27	10.9
Painful sexual intercourse	37	15.0
Burning pain during urination	70	28.3
Irregular periods	52	21.1
Itchiness in genital area	85	34.4
Other (vaginal burning (3), dysmenorrhoea (3), urine incontinence (3), painful stitches from prior c-section (2), lower abdominal pain (1), reduced libido (1), rash (1))	14	5.7
No response	0	0.0
**The last time you had any of these symptoms above, where did you get treatment?* (n = 151, m = 0)**		
I did not get treatment	68	45.0

Health clinic in camp (BRF, CMA, MDM, EODY)	66	43.7
Health clinic out of camp (MSF)	54	35.8
Referral hospital	6	4.0
Pharmacy	1	0.7
Traditional medication	4	2.6
Other (medication from home country)	1	0.7
No response	0	0.0
**ABORTION**		
**Sometimes women are worried they are pregnant or get pregnant when they do not want to be and they do something to remove the pregnancy. With your permission, I am going to ask you some questions about this topic. Are you feeling comfortable with continuing? (n = 247, m = 0)**		
Yes	217	87.9
No	30	12.1
**Where do you go to get information about issues related to removing pregnancy?* (n = 216, m = 1)**		
I did not get information	184	85.2
Healthcare professionals	20	9.3
Trained volunteers or counsellors from NGOs	10	4.6
Community leaders or representatives	0	0.0
Religious or spiritual leaders	0	0.0
Female family members or trusted female friends	3	1.4
Pamphlets or educational materials provided in the camp	0	0.0
Online resources or websites	1	0.5
Other (school)	1	0.5
No response	4	1.9
**Where and from whom would you PREFER to receive information about removing pregnancy?* (n = 216, m = 1)**		
I prefer not to receive information	41	19.0
Healthcare professionals	154	71.3
Trained volunteers or counsellors from NGOs	32	14.8
Community leaders or representatives	0	0.0
Religious or spiritual leaders	0	0.0
Female family members or trusted female friends	7	3.2
Pamphlets or educational materials provided in the camp	2	0.9
Online resources or websites	12	5.6
Other	0	0.0
No response	5	2.3
**Since arriving in camp, have you done something to remove a pregnancy when you were pregnant or worried you were pregnant? (n = 217, m = 0)**		
Yes	2	0.9
No	212	97.7
No response	3	1.4
**When you ended your last pregnancy in camp, how many weeks pregnant were you? (n = 2, m = 0)**		
Number of Weeks (n = 1)	6	50.0
No response	1	50.0
**Which of the following methods did you use to remove your pregnancy? (n = 2, m = 0)***		
Surgery from a healthcare professional	0	0.0
Pills from a healthcare professional	2	100
Pills I bought myself without consulting a healthcare professional	0	0.0
Traditional medication	0	0.0
Insert materials into the vagina	0	0.0
Other	0	0.0
No response	0	0.0
**Where did you receive services to remove your last pregnancy in camp? (n = 2, m = 0)***		
I did not receive services	0	0.0
Health clinic in camp (BRF, CMA, MDM, EODY)	1	50.0
Health clinic out of camp (MSF)	1	50.0
Referral hospital	0	0.0

Other	0	0.0
No response	0	0.0
I did not receive services	0	0.0
**While using the above-mentioned methods to remove your pregnancy, did you experience any of the following complications or symptoms?* (n = 2, m = 0)**		
I did not have any complications	0	0.0
Heavy pain or cramping	2	100.0
Heavy bleeding	1	50.0
High fever	0	0.0
Infection	0	0.0
Other	0	0.0
No response	0	0.0
**Where did you seek care for the above-mentioned complications associated with removing your pregnancy?* (n = 2, m = 0)**		
I did not seek any care	1	50.0
Health clinic in camp (BRF, CMA, MDM, EODY)	0	0.0
Health clinic out of camp (MSF)	1	50.0
Referral hospital	0	0.0
Other	0	0.0
No response	0	0.0
I did not seek any care	0	0.0
**FEMALE GENITAL MUTILATION/CUTTING**		
**In a number of countries, there is a practice in which a girl may have part or all of her genitals cut. With your permission, I am going to ask you some questions about this topic. Are you feeling comfortable with continuing? (n = 247, m = 0)**		
Yes	215	87.0
No	32	13.0
**Where do you go to get information about issues related to female genital cutting or mutilation?* (n = 215, m = 0)**		
I did not get information	195	90.7
Healthcare professionals	3	1.4
Trained volunteers or counsellors from NGOs	9	4.2
Community leaders or representatives	0	0.0
Religious or spiritual leaders	0	0.0
Female family members or trusted female friends	6	2.8
Pamphlets or educational materials provided in the camp	0	0.0
Online resources or websites	5	2.3
Other (school (1), unknown (1))	2	0.9
No response	0	0.0
**Where and from whom would you PREFER to receive information about female genital cutting or mutilation?* (n = 215, m = 0)**		
I prefer not to receive information	57	26.5
Healthcare professionals	129	60.0
Trained volunteers or counsellors from NGOs	38	17.7
Community leaders or representatives	2	0.9
Religious or spiritual leaders	3	1.4
Female family members or trusted female friends	5	2.3
Pamphlets or educational materials provided in the camp	5	2.3
Online resources or websites	20	9.3
Other (no preference)	1	0.5
No response	0	0.0
**Have you experienced any of the following forms of female genital mutilation in your life:* (n = 215, m = 0)**		
I have never experienced any of the following	139	64.7
Clitoris partially or fully cut	44	20.5
Clitoris and vulva partially or fully cut	25	11.6
Genital area fully or partially sewn closed	15	7.0
Pricking, piercing, scraping or burning of the genital area	7	3.3
Other (vaginal enlargement (1), unsure (5))	6	2.8
No response	0	0.0

**How old were you when this occurred? (n = 76, m = 0)**		
Median Age in years (n = 72)	6.5 (IQR 4.0 – 8.3, range 0 - 25)	94.7
No response	4	5.3
**Did you seek healthcare services for FGM/C while living here in the camp?* (n = 76, m = 0)**		
I did not seek FGM/C-related treatment	68	89.5
Mental health support	2	2.6
Treatment for infection	4	5.3
Treatment for bleeding	1	1.3
Treatment for urinary issues	2	2.6
Surgery	1	1.3
Other (vulnerability certificate (1), sought care but did not access (1), sought treatment for painful intercourse/reduced libido/dysmenorrhoea (1))	3	3.9
No response	1	1.3
**Where did you seek care for the above-mentioned FGM/C-related healthcare services?* (n = 7, m = 1)** *NOTE: All respondents except those who answered 'I did not seek FGM/C-related treatment' were asked this question.*		
Health clinic in camp (BRF, CMA, MDM, EODY)	2	25.0
Health clinic out of camp (MSF)	6	75.0
Referral hospital	0	0.0
Other	0	0.0
No response	0	0.0
**GENDER-BASED VIOLENCE**		
**Women can sometimes experience emotional, physical or sexual violence. With your permission, I am going to ask you some questions about this topic. Are you feeling comfortable with continuing? This is the last section of the interview and I would like to remind you that you are free to stop if you want. (n = 247, m = 0)**		
Yes	244	98.8
No	3	1.2
No response	0	0.0
**Did you get married before the age of 18 years old? (n = 243, m = 1)**		
Yes	67	27.6
No	176	72.4
No response	0	0.0
**Please note the following locations where someone shouted at, insulted or threatened you (n = 244, m = 0)***		
I have never been shouted at, insulted or threatened	63	25.8
In camp now	35	14.3
Any previous camp	21	8.6
During a pushback	45	18.4
In a detention centre	30	12.3
While traveling by road/boat	49	20.1
In my home country	119	48.8
Other (unspecified place in Türkiye (10), unspecified place in Ethiopia (2), unspecified place in Lebanon (1), unspecified place in Somalia (1), unspecified place in Saudi Arabia (1), unspecified place in Iran (1), Greek asylum office (1), supermarket Greece (1), hospital in unspecified country (1), NGO in Greece (1), hospital and police station in Türkiye (1), asylum office in Türkiye (1), workplace in Türkiye (1), workplace in unspecified country (1))	24	9.8
No response	1	0.4
**Who did this to you? (n = 180, m = 1)***		
Military	36	20.0
Police or prison guard	88	48.9
Medical staff	5	2.8
Humanitarian relief worker	1	0.6
Neighbour/community member	51	28.3
Fellow refugee/internally displaced persons	18	10.0
Family member	46	25.6
Partner/husband	36	20.0

Other (smuggler (15), employer (5), case worker (5), Taliban (4), Al Qaeda (3), unknown stranger (3), robber (1), hospital staff (1), security personnel (1), camp staff (1))	39	21.7
No response	2	1.1
**Please note the following locations where someone physically hurt you, such as beating or kicking: (n = 244, m = 0)***		
I have never been physically hurt	114	46.7
In my home country	88	36.1
During a pushback	24	9.8
While traveling by road/boat	21	8.6
Other (unspecified place in Türkiye (7), unspecified place in Iran (4), hospital in Türkiye (1), unspecified place in Ethiopia (1), unspecified place in South Africa (1), unspecified place in Saudi Arabia (1), unspecified place in Somalia (1), unspecified place in Afghanistan (1))	17	7.0
In a detention centre	10	4.1
In camp now	7	2.9
Any previous camp	4	1.6
No response	3	1.2
**Who did this to you? (n = 127, m = 3)***		
Military	19	15.0
Police or prison guard	36	28.3
Medical staff	1	0.8
Humanitarian relief worker	0	0.0
Neighbour/community member	33	26.0
Fellow refugee/internally displaced persons	6	4.7
Family member	42	33.1
Partner/husband	40	31.5
Other (smuggler (6), unknown stranger (3), bandits (2), Al Qaeda (2), employer (1), Taliban (1))	15	11.8
No response	0	0.0
**Where did you seek care for the physical abuse? (n = 126, m = 4)***		
I did not seek care	101	80.2
I got care before arriving in camp	11	8.7
Health clinic in camp (BRF, CMA, MDM, EODY)	5	4.0
Health clinic out of camp (MSF)	8	6.3
Referral hospital	3	2.4
Other (psychologist with NGO (3))	3	2.4
No response	1	0.8
**Please note the following locations where someone sexually abused you: (n = 244, m = 0)***		
I have never been sexually abused	156	63.9
In my home country	55	22.5
While traveling by road/boat	17	7.0
During a pushback	9	3.7
In a detention centre	4	1.6
In camp now	3	1.2
Any previous camp	2	0.8
Other (unspecified place in Türkiye (7), unspecified place in Ethiopia (2), unspecified place in Iran (2), unspecified place in Lebanon (1), unspecified place in South Africa (1), unspecified place in Saudi Arabia (1), unspecified place (1))	15	6.1
No response	1	0.4
**Who did this to you? (n = 87, m = 1)***		
Military	6	6.9
Police or prison guard	14	16.1
Medical staff	0	0.0
Humanitarian relief worker	0	0.0
Neighbour/community member	27	31.0
Fellow refugee/internally displaced persons	2	2.3

Family member	9	10.3
Partner/husband	26	29.9
Other (unknown stranger (4), smuggler (6), employer (4), landlord (2), bandits (2), bus driver (1), tribal head (1), unknown (4))	24	27.6
No response	0	0.0
**Where did you seek care for the sexual abuse? (n = 87, m = 1)***		
I did not seek care	55	63.2
I got care before arriving in camp	3	3.4
Health clinic in camp (BRF, CMA, MDM, EODY)	6	6.9
Health clinic out of camp (MSF)	24	27.6
Referral hospital	0	0.0
Other (psychologist (5), vulnerability case support (1))	6	6.9
No response	0	0.0
