# Supplementary material for: Sexual and reproductive health needs of refugee women on Lesbos, Greece: a participatory cross-sectional study
Source: BMJ Glob Health. 2026 Jun 28;11(6):e019240. doi: 10.1136/bmjgh-2025-019240 (PMC13311758; doi:10.1136/bmjgh-2025-019240)
Supplement: online supplemental file 4 [file bmjgh-11-6-s010.pdf]

Definition of Variables

Demographics
Age was reported as a continuous variable and recategorized for analysis into 3 groups: 15-24 years, 25-34 years, and ≥35 years. Country of origin was reported by country name and recategorized for analysis into the following groups with frequencies above 5: Afghanistan, Eritrea, Somalia, Yemen, Palestine, Democratic Republic of Congo, Syria, and Other. Religion was categorized into three groups: Muslim, Christian, and Other.

Socioeconomic Factors and Living Arrangements
Education status of participants was recorded as the highest level of education they had completed: never attended school, primary, secondary, post-secondary, or other. Part of the LBGTQI community was recorded with two possible responses: yes or no. Living with a partner in camp was also recorded with two possible responses: yes or no. Number of children was reported as a continuous variable.

Displacement History
Number of years displaced was calculated by subtracting 2023 from the respondent's reported year of displacement. Months in camp was reported as a continuous variable. Experienced pushbacks before arriving was recorded with two possible responses: yes or no. The number of pushbacks experienced was reported as a continuous variable. For those who had experienced a pushback, the median was calculated.

Asylum Process
The status of asylum requests was recorded as either receiving a negative response or not, categorized as yes or no.

Healthcare Access
Frequency of being denied medical care because of legal status was recorded as always, often, sometimes, rarely, or never.

Information
Sources of received and preferred information was recorded as follows: I did not get information/I do not prefer to receive information, healthcare professionals, trained volunteers or counselors, community leaders or representatives, religious or spiritual leaders, female family members or trusted female friends, pamphlets or education material provided in camp, online resources or websites, other or no response. Multiple answers were possible and answers were analysed as a percentage of the total number of respondents who answered the question.

Maternal Health
Length of pregnancy was recorded in months. For those who answered in weeks, weeks were converted to months in decimal. Number of children was recorded as a continuous variable, with 108 women who did not have any children included. Our survey did not specify whether children were alive nor whether they currently resided with the woman. We defined single mothers as those who selected "No" to "Are you currently living with a partner or husband?" and indicated that they had children. Seven women who selected "I did not go to the health centre" were included in the "Median Number of Health Centre Visits" calculation for antenatal care. "Median months baby was breastfed" calculation excluded the women who were still breastfeeding.

Contraception Preferences
Preferred methods to prevent pregnancy was recorded as following: no preference, the pill (oral contraceptives), Intra-Uterine Device (IUD), male condoms, female condoms, implants, injectables, emergency hormonal contraception, tubal ligation, rhythm/calendar method, withdrawal, abstinence, traditional medication, and other. Modern contraception methods were considered to include the pill (oral contraceptives), IUD, male condoms, female condoms, implants, injectables, and tubal ligation. Based on these methods reported, preference for modern contraception was recategorized into two groups: prefer modern contraception and do not prefer modern contraception.

Contraception Access
Methods accessed to prevent pregnancy were detailed as follows: no methods, the pill (oral contraceptives), IUD, male condoms, female condoms, implants, injectables, emergency hormonal contraception, tubal ligation, rhythm/calendar method, withdrawal, abstinence, traditional medication, and other. Modern contraception methods were considered to include the pill (oral contraceptives), IUD, male condoms, female condoms, implants, injectables, and tubal ligation. Based on these methods reported, access to modern contraceptives was recategorized into two groups: accessed and did not access.

Menstrual Health
Menstrual health questions pertained to the respondent's last menstrual period. Menstrual materials were detailed as follows: single use menstrual pads/liners, reusable menstrual pads, tampons, menstrual cup, cloth or cotton wool, toilet paper, underwear only (non-absorbent), nothing, and other. Ability to relieve pain was recorded as: I did not have pain, always, often, sometimes, rarely, or never. Options for how pain was managed included: home remedies or self-care techniques, buying medication, going to the doctor, or other. Respondents were asked whether the place where they changed their menstrual materials in the camp was clean, private, and had soap and water. They could answer "yes" or "no" to each condition or indicate that they did not use the camp toilet during their last menstrual period.

Gynecological Symptoms Prevalence
Gynecological symptoms were recorded by combining responses from two different survey questions. The first question asked participants: "Since arriving in camp, have you had any of the following symptoms?" with options including: I did not have any symptoms, bleeding in between periods, painful sexual intercourse, burning pain during urination, irregular periods, itchiness in genital area, and other. The second question asked: "Have you had any of the following symptoms since arriving in camp?" with options including: I did not have any symptoms, genital sores/ulcers, unusual genital discharge, hot, swollen, red genital area, and other. Symptoms were recategorized as experienced or did not experience. Specific symptoms included no symptoms, genital sores/ulcers, unusual genital discharge, hot, swollen, red genital area, bleeding in between periods, painful sexual intercourse, burning pain during urination, irregular periods, itchiness in genital area, other, and no response.

Gynecological Symptoms Care Access
Access to care for gynecological symptoms was determined by combining responses from two different survey questions. Each question asked participants: "The last time you had any of these symptoms above, where did you get treatment?" for gynecological symptoms and STI symptoms separately. Options were reported as: I did not get treatment, health clinic in camp, health clinic out of camp, referral hospital, pharmacy, traditional medication, and other. Based on these responses, treatment locations were recorded as no treatment, health clinic in camp, health clinic out of camp, referral hospital, pharmacy, traditional medication, other, and no response. Access to care was recategorized as accessed care and did not access care based on the combined reported locations of access.

Female Genital Mutilation/ Cutting Care Access
To determine whether women successfully accessed care we asked, "Did you seek healthcare services for FGM/C while living here in the camp?", however, in retrospect realise that this question does not account for women who sought services but did not receive them. Three respondents went to clinics to treat FGM-related symptoms but did not receive care. These women were not counted in for the question addressing "Where did you seek care?"

Gender-Based Violence Prevalence
GBV was recorded based on physical harm and sexual abuse. Responses to where respondents experienced physical or sexual violence were combined from two different survey questions to determine the prevalence of GBV. The locations for physical harm and sexual abuse both included: in camp now, any previous camp, during a pushback, in a detention center, while traveling by road/boat, in home country, and other. By combining these responses, GBV was recategorized into two groups: experienced or did not experience.

Gender-Based Violence Care Access
Access to care for GBV was recorded based on care for physical harm and sexual abuse. Responses to where respondents accessed care from physical or sexual violence were combined from two different survey questions to determine the rates of access to care for GBV. The locations for accessing care for physical harm and sexual abuse both included: no care, care before arriving in camp, health clinic in camp, health clinic out of camp, referral hospital, and other. By combining these responses, access to GBV care was recategorized into two groups: accessed care or did not access care.
