# Supplementary material for: Sexual and reproductive health needs of refugee women on Lesbos, Greece: a participatory cross-sectional study
Source: BMJ Glob Health. 2026 Jun 28;11(6):e019240. doi: 10.1136/bmjgh-2025-019240 (PMC13311758; doi:10.1136/bmjgh-2025-019240)
Supplement: online supplemental file 5 [file bmjgh-11-6-s011.docx]

**Bivariate analyses**

Note that Syria, Sierra Leone, Sudan, Ethiopia, Cameroon, Iraq, Uganda, Iran, Guinea, Liberia and Tajikistan are excluded in the below table due to sample size. Similarly, respondents could also indicate they had no contraception preference. This explains why sums do not consistently add up to 100%. Contraception preference was asked to all respondents in the sample who consented to the section (n=242). Contraceptive use was asked to respondents who said they wanted to prevent pregnancy (n=178). Experience of FGM/C was assessed among respondents who consented to that section (n = 215); after excluding missing responses to the FGM/C item, the analytic sample was n=197.

| **Country of origin, n (%)** | **Preferred modern contraceptives** | **Did not prefer modern contraceptives** | **P value** | **Used modern contraceptives** | **Did not use modern contraceptives** | **P value** | **Experienced FGM/C** | **Did not experience FGM/C** | **P value** |
| --- | --- | --- | --- | --- | --- | --- | --- | --- | --- |
| **Afghanistan** | 50 (61%) | 32 (39%) | 0.070 | 34 (56.7%) | 26 (43.3%) | **<0.001** | 0 (0%) | 73 (100%) | **<0.001** |
| **Eritrea** | 41 (62.1%) | 25 (37.9%) |  | 6 (12%) | 44 (88%) |  | 26 (40%) | 39 (60%) |  |
| **Somalia** | 17 (56.7%) | 13 (43.3%) |  | 0 (0%) | 23 (100%) |  | 28 (96.6%) | 1 (3.4%) |  |
| **Yemen** | 5 (29.4%) | 12 (70.6%) |  | 1 (6.7%) | 14 (93.3%) |  | 13 (76.5%) | 4 (23.5%) |  |
| **Palestine** | 4 (33.3%) | 8 (66.7%) |  | 0 (0%) | 7 (100%) |  | 0 (0%) | 8 (100%) |  |
| **Democratic Republic of Congo** | 5 (41.7%) | 7 (58.3%) |  | 1 (12.5%) | 7 (87.5%) |  | 1 (20%) | 4 (80%) |  |
